# Inequalities in Access and Utilization of Maternal, Newborn and Child Health Services in sub-Saharan Africa: A Special Focus on Urban Settings

**DOI:** 10.1007/s10995-021-03250-z

**Published:** 2021-10-15

**Authors:** E. M. Sidze, F. M. Wekesah, L. Kisia, A. Abajobir

**Affiliations:** African Population and Health Research Center (APHRC), APHRC Campus, 2nd Floor, Manga Close off Kirawa Road, P.O. Box 10787-00100, Nairobi, Kenya

**Keywords:** Maternal and child health, Equity, Universal health coverage, sub-Saharan Africa, Systematic review

## Abstract

**Objectives:**

The aim of this paper is to share the results of a systematic review on the state of inequalities in access to and utilization of maternal, newborn and child health (MNCH) services in the sub-Saharan African region. The focus of the review was on urban settings where growing needs and challenges have been registered over the past few years due to rapid increase in urban populations and urban slums.

**Methods:**

The review was conducted according to Preferred Reporting Items for Systematic Reviews and Meta-analyses (PRISMA) guidelines. Studies published in English between 2000 and 2019 were included. A narrative synthesis of both qualitative and quantitative data was undertaken. The record for registration in PROSPERO was CRD42019122066.

**Results:**

The review highlights a great variation in MNCH services utilization across urban sub-Saharan Africa (SSA). The main aspects of vulnerability to unequal and poor MNCH services utilization in urban settings of the region include poverty, low level of education, unemployment, lower socioeconomic status and poor livelihoods, younger maternal age, low social integration and social support, socio-cultural taboos, residing in slums, and being displaced, refugee, or migrant. At the health system level, persistent inequalities are associated with distance to health facility, availability of quality services and discriminating attitudes from health care personnel.

**Conclusion:**

Context-specific intervention programs that aim at resolving the identified barriers to access and use MNCH services, particularly for the most vulnerable segments of urban populations, are essential to improve the overall health of the region and universal health coverage (UHC) targets.

**Supplementary Information:**

The online version contains supplementary material available at 10.1007/s10995-021-03250-z.

## Significance Statement

*What is already know on this subject*? The new face of urban poverty in the SSA region is linked to adverse health outcomes for women and children. Comprehensive approaches to improve MNCH outcomes in the region therefore require identifying and responding to barriers to access and utilization of quality services, focusing particularly on poor populations living in urban settings.

*What this study adds*? This systematic review provides up-to-date and program-relevant information on the state of inequalities among the urban population in SSA.

## Introduction

Efforts have been made globally to improve maternal, newborn and child health (MNCH). According to the World Health Organization (2019), maternal mortality ratio dropped by 38% globally between 2000 and 2017. Several sub-Saharan Africa (SSA) countries have halved their maternal mortality since 2000. However, low-income countries including the SSA region continues to contribute to more than half of all the maternal deaths in low- and middle-income countries (LMICs) (World Health Organization, 2019). In 2017, the maternal mortality ratio (MMR) in LMICs, including countries from SSA, was 462 per 100,000 live births but it was 11 per 100,000 live births in developed regions (World Health Organization, 2019). Furthermore, SSA countries report the highest neonatal mortality rate (NMR) in the world with about 28 neonates per 1000 livebirths dying within the first 28 days of life in 2017. From 1990 to 2017, the decline in NMR in SSA (East, West, central and south Africa, excluding North Africa) was low (40%) compared to that of high income countries (55%) (Hug et al., 2019). In 2015, the mortality rate of children under the age of 5 was the highest in SSA (83 per 1000 livebirths), sharing 50% of under 5 deaths worldwide. This rate is significantly higher as compared to high income counties (7 per 1000 live births) (Hug et al., 2019).

The slow improvement in MNCH outcomes in SSA can be attributed to discrepancies in care seeking behaviors and the low utilization of health services (Hug et al., 2019). For instance, only 48% of women in SSA give birth with the assistance of skilled personnel compared to 72% of women globally (Amouzou et al., 2017). Countries in SSA continue also to display persistent inequalities in access to, and utilization of, quality services with significant differences in access between poor and non-poor populations (Faye et al., 2020; Sidze et al., 2020; Wehrmeister et al., 2020). Wealth-related inequalities remain high, with socioeconomic status and place of residence (urban or rural) being the main predictors of differences in the utilization of MNCH services and influence maternal and child health outcomes (Wehrmeister et al., 2020). A retrospective review of survey data from 54 LMICs revealed that skilled-birth attendance coverage and 4 or more ANC visits were the least equitable interventions. The mean skilled-birth attendance coverage for the 54 countries was lower in the poorest quintile (32%) compared with the richest quantile (84%) (Barros et al., 2012).

Access to quality MNCH services is more challenging for poor populations living in urban settings, in particular those settling in the slums. The growth of urban slums which is characterized by overcrowding, social and economic marginalization, poor environmental conditions, and insecurity has intensified since the 1990s in the SSA region and evidence shows that urban health and social indicators have either deteriorated significantly or even reversed in favor of rural areas (World Health Organization & U. N. Habitat, 2016). The new face of urban poverty in SSA is linked to adverse MNCH outcomes. In Nigeria, for instance, the MMR among women living in the urban slums of Lagos was two times higher than the ratio estimated for the entire Lagos State (Anastasi et al., 2017). A similar situation is observed in the urban slums of Nairobi (African Population and Health Research Center (APHRC) 2014; Kimani-Murage et al., 2014; Ziraba et al., 2009). Comprehensive approaches to improve MNCH outcomes in SSA will therefore require identifying, addressing, and responding to barriers to access and utilization of quality services, while focusing particularly on populations living in urban settings. This systematic review thus provides up-to-date information on the state of access to MNCH services and observed inequalities among the urban population in SSA.

## Methods

This review was conducted according to the recommendations outlined in the Preferred Reporting Items for Systematic Reviews and Meta-analyses (PRISMA) statement (Liberati et al., 2009). The review was registered with PROSPERO under the record number CRD42019122066.

### Search Strategy

We searched PubMed, Embase, Scopus, Africa Index Medicus (AIM), Africa Journals Online (AJOL), and Google Scholar databases to retrieve relevant articles on primary studies conducted in SSA, using pre-defined search (Title/Abstract) and indexing terms (MeSH). Keywords and MeSH terms and their combinations used in the searches included: “health”, “socio-economic status”, “wealth”, “equity”, “vulnerability”, “urban poor”, “migrant”, “accessibility”, “availability”, “affordability”, “maternal health services”, “child health services”, and “breastfeeding”. Reference lists of selected articles were searched for additional relevant articles. The strategy is provided as a supplementary file (Supplementary 1).

### Participants

We included studies conducted at the population level on access, coverage and inequities in maternal, newborn and child health services, and at health care facilities on health care system assessments in urban SSA.

### Inclusion Criteria

We included studies that were published in English, and in peer-reviewed journals between 2000 (representing the inception of the Millennium Development Goals) to 2019. Papers were from primary research of any design and methodology, conducted in any of the countries in SSA, or at the regional level using data from primary research, demographic and health surveys (DHS), or demographic and health surveillance systems (DHSS). Studies that were carried out among SSA populations living in western countries were excluded. Systematic reviews, case reports, review articles, editorials and letters to the editor were also excluded.

### Definition of Terms/Concepts

#### MNCH Services

MNCH services included all services along the continuum of care (Shibanuma et al., 2018). These services included care received by women and children at prenatal, delivery and postnatal periods.

#### Health Inequalities

Health inequalities refer to “systematic, avoidable and unfair differences in health outcomes that can be observed between populations, between social groups within the same population or as a gradient across a population ranked by social position” (McCartney et al., 2019). Therefore, the inequalities in access to and utilization of MNCH services in this review were assessed by looking at how access and coverage of services differ among various sub-populations in urban SSA with respect to social and/or economic obstacles or other characteristics linked to discrimination or exclusion. These factors include, for instance, ethnic group, religion, gender, age, HIV status, geographic location, and status in the country of residence (migrant versus residents). The SSA region was classified based on the United Nations classification of countries (United Nations, 2014).

### Data Extraction

Two reviewers (FW and a consultant) conducted data extraction from the identified studies. A data extraction form was developed from published resources. The following study characteristics were documented from each of the selected articles: author(s) and country, year of publication, study setting, study design, study population, age range of participants, study type (qualitative, quantitative or mixed methods), and study findings (coverage, access and utilization of MNCH services). EMS provided overall guidance on the extraction process. EMS and AA drafted the manuscript.

### Quality Assessment

Two reviewers (FW and a consultant) assessed the quality of included studies. Randomized and non-randomized control trials were assessed using the revised Cochrane risk of bias tools for randomized trials (ROB-2) (Higgins et al., 2018). The quality of other quantitative studies were assessed based on National Institute of Health (NIH) Quality Assessment Tool for Observational Cohort and Cross-Sectional Studies (2014). This form appraised the reliability, validity and generalizability of the quantitative studies. The NIH quality assessment tool uses 13 criteria to assess and rate the quality of studies. This included the research question, study population, sample size estimation, exposure and outcome assessment, loss to follow-up, and statistical analysis. General guidance is provided for determining the overall quality of the studies and to grade their level of quality as good, fair, or poor.

Qualitative studies were appraised using the Critical Appraisal Skill Programme (CASP) tool (2013). The CASP tool has 10 items that look at the relevance and clarity of research goals, appropriateness of the research design and methodology in addressing the research question, recruitment strategies, data collection, data analysis, findings, ethical consideration, and value of the research. Questions attached to these items enable critical self-reflection about biases and assess the extent to which findings from the study could be transferred to other settings or groups. The quality assessment and criteria are available as Supplementary File 2.

### Synthesis of Findings

A quantitative and non-quantitative synthesis was conducted and a tabular evidence profile for each study was prepared. The findings from each of the selected articles were reviewed and the evidence weighed by considering the number of articles, study design and associated sample size. Findings of the study were reported in a narrative synthesis. Findings from qualitative articles were integrated with those from the quantitative studies based on similar themes or topics. Due to the heterogeneity in outcomes, data were not pooled to conduct a meta-analysis.

## Results

### Study Selection

**Fig. 1:**
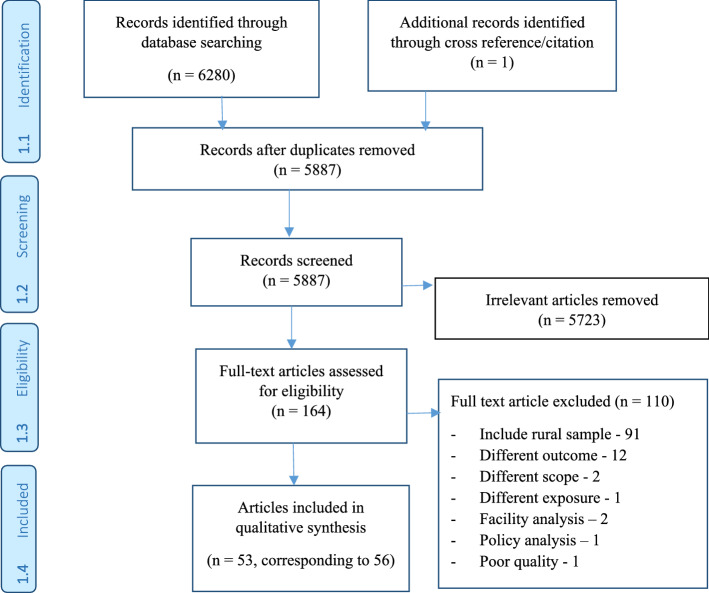
Flow chart for study inclusion and exclusion steps

The systematic search of several databases and cross-referencing resulted in a total of 6280 research articles. After removing duplicate records, a total of 5887 articles were screened based on their titles and abstracts of which, 5723 articles were excluded as they were irrelevant for the current review. We assessed a further 164 full-text articles using a priori eligibility criteria. From these, 110 articles were excluded due to various reasons such as their inclusion of a rural sample (91 articles), assessing a different outcome from the focus of our review (12 articles), difference in scope of the study (e.g., population level, facility and/or policy analyses) and exposure (seven articles). Finally, 53 research articles from 11 SSA countries were included in this systematic review. The selection process is summarized in Fig. 1.

### Study Characteristics

Majority of the studies included in the review were conducted in East Africa (Adane et al., 2017; Babirye et al., 2014; Bayou et al., 2016; Bayu et al., 2015; Belayneh et al., 2014; Bellows et al., 2012; Birungi et al., 2011; Chege et al., 2002; Demilew, 2017; Egondi et al., 2015; Fotso et al., 2008, 2009; Izugbara et al., 2009; Kawakatsu et al., 2015; Kimani-Murage et al., 2015, 2016; Kuwawenaruwa et al., 2016; Machira & Palamulen, 2017; Mekonnen & Mekonnen, 2003; Mirkuzie, 2014; Mustapha et al., 2018; Mutua et al., 2011; Ndimbii et al., 2018; Ng’anjo Phiri et al., 2014; Ochola et al., 2013; Owiti et al., 2018; Sasaki et al., 2010, 2011; Taffa & Chepngeno, 2005; Tann et al., 2007; Wakwoya et al., 2016; Westheimer et al., 2004; Wilunda et al., 2016; Yaya et al., 2018), followed by West (Abimbola et al., 2016; Adewuyi et al., 2017, 2018; Aidam et al., 2005a, 2005b; Anastasi et al., 2017; Antai, 2010; Asundep et al., 2013; Fatiregun & Okoro, 2012; Idowu et al., 2017; Jennings et al., 2017; Nwameme et al., 2014; Olusanya et al., 2010; Woldeghebriel et al., 2017) and Southern (Kibiribiri et al., 2016; Ngandu et al., 2017) Africa; 17 (30.9%) studies were conducted in Kenya (Bellows et al., 2012; Birungi et al., 2011; Chege et al., 2002; Egondi et al., 2015; Fotso et al., 2008, 2009; Izugbara et al., 2009; Kawakatsu et al., 2015; Kimani-Murage et al., 2015, 2016; Mutua et al., 2011; Ndimbii et al., 2018; Ng’anjo Phiri et al., 2014; Ochola et al., 2013; Owiti et al., 2018; Taffa & Chepngeno, 2005), followed by 9 (16.4%) studies each of which were conducted in Ethiopia (Adane et al., 2017; Bayou et al., 2016; Bayu et al., 2015; Belayneh et al., 2014; Demilew, 2017; Mekonnen & Mekonnen, 2003; Mirkuzie, 2014; Wakwoya et al., 2016; Yaya et al., 2018) and Nigeria (Abimbola et al., 2016; Adewuyi et al., 2017, 2018; Anastasi et al., 2017; Antai, 2010; Fatiregun & Okoro, 2012; Idowu et al., 2017; Olusanya et al., 2010). Similarly, 6 (10.9%) studies were conducted in Ghana (Aidam et al., 2005a, 2005b; Asundep et al., 2013; Nwameme et al., 2014; Woldeghebriel et al., 2017). Other countries where the studies were carried out include Uganda (Babirye et al., 2014; Mustapha et al., 2018; Tann et al., 2007), Tanzania (Kuwawenaruwa et al., 2016; Ng’anjo Phiri et al., 2014; Westheimer et al., 2004), Zambia (Ng’anjo Phiri et al., 2014; Sasaki et al., 2010, 2011), South Africa (Kibiribiri et al., 2016; Ngandu et al., 2017), Malawi (Machira & Palamulen, 2017), and South Sudan (Wilunda et al., 2016). Three of the included articles were multi-country studies conducted in Kenya, Tanzania, and Zambia (Ng’anjo Phiri et al., 2014), Kenya and Burkina Faso (Rossier et al., 2014), and Ghana and Nigeria (Jennings et al., 2017).

These studies were published from 2002 to 2019, of which 13 (25.5%) were published before 2010 (Aidam et al., 2005a, 2005b; Antai, 2010; Chege et al., 2002; Fotso et al., 2008, 2009; Izugbara et al., 2009; Mekonnen & Mekonnen, 2003; Olusanya et al., 2010; Sasaki et al., 2010; Taffa & Chepngeno, 2005; Tann et al., 2007; Westheimer et al., 2004), and 38 (74.5%) studies published after 2011 (Abimbola et al., 2016; Adane et al., 2017; Adewuyi et al., 2017, 2018; Anastasi et al., 2017; Asundep et al., 2013; Babirye et al., 2014; Bayou et al., 2016; Bayu et al., 2015; Belayneh et al., 2014; Bellows et al., 2012; Birungi et al., 2011; Demilew, 2017; Fatiregun & Okoro, 2012; Idowu et al., 2017; Kawakatsu et al., 2015; Kibiribiri et al., 2016; Kimani-Murage et al., 2015, 2016; Kuwawenaruwa et al., 2016; Machira & Palamulen, 2017; Mirkuzie, 2014; Mustapha et al., 2018; Mutua et al., 2011; Ndimbii et al., 2018; Ng’anjo Phiri et al., 2014; Ngandu et al., 2017; Nwameme et al., 2014; Ochola et al., 2013; Owiti et al., 2018; Rossier et al., 2014; Sasaki et al., 2011; Wakwoya et al., 2016; Wilunda et al., 2016; Woldeghebriel et al., 2017; Yaya et al., 2018). Twenty-three of the studies (45.1%) were conducted in general urban settings (Adane et al., 2017; Aidam et al., 2005a, 2005b; Anastasi et al., 2017; Asundep et al., 2013; Babirye et al., 2014; Bayou et al., 2016; Bayu et al., 2015; Belayneh et al., 2014; Demilew, 2017; Fatiregun & Okoro, 2012; Idowu et al., 2017; Kawakatsu et al., 2015; Kibiribiri et al., 2016; Mirkuzie, 2014; Mustapha et al., 2018; Mutua et al., 2011; Nwameme et al., 2014; Olusanya et al., 2010; Owiti et al., 2018; Tann et al., 2007; Westheimer et al., 2004; Woldeghebriel et al., 2017), while 11 (21.6%) were carried out in urban slum areas (Bellows et al., 2012; Chege et al., 2002; Egondi et al., 2015; Fotso et al., 2008, 2009; Izugbara et al., 2009; Kimani-Murage et al., 2015, 2016; Ochola et al., 2013; Rossier et al., 2014; Taffa & Chepngeno, 2005).

In terms of research methods used, 47 (88.7%) studies were quantitative (Abimbola et al., 2016; Adane et al., 2017; Adewuyi et al., 2017, 2018; Aidam et al., 2005a, 2005b; Anastasi et al., 2017; Antai, 2010; Asundep et al., 2013; Bayou et al., 2016; Bayu et al., 2015; Belayneh et al., 2014; Bellows et al., 2012; Birungi et al., 2011; Demilew, 2017; Egondi et al., 2015; Fatiregun & Okoro, 2012; Fotso et al., 2008, 2009; Idowu et al., 2017; Jennings et al., 2017; Kawakatsu et al., 2015; Kimani-Murage et al., 2016; Machira & Palamulen, 2017; Mekonnen & Mekonnen, 2003; Mutua et al., 2011; Ng’anjo Phiri et al., 2014; Ngandu et al., 2017; Nwameme et al., 2014; Ochola et al., 2013; Olusanya et al., 2010; Owiti et al., 2018; Rossier et al., 2014; Sasaki et al., 2010, 2011; Taffa & Chepngeno, 2005; Tann et al., 2007; Wakwoya et al., 2016; Westheimer et al., 2004; Woldeghebriel et al., 2017; Yaya et al., 2018) and/or mixed-methods (Babirye et al., 2014; Chege et al., 2002; Kibiribiri et al., 2016; Kuwawenaruwa et al., 2016; Mustapha et al., 2018), and 4 (7.8%) were qualitative (Izugbara et al., 2009; Kimani-Murage et al., 2015; Mirkuzie, 2014; Ndimbii et al., 2018; Wilunda et al., 2016). Majority of the studies were cross-sectional (Abimbola et al., 2016; Aidam et al., 2005a; Asundep et al., 2013; Babirye et al., 2014; Bayou et al., 2016; Belayneh et al., 2014; Bellows et al., 2012; Birungi et al., 2011; Chege et al., 2002; Demilew, 2017; Fatiregun & Okoro, 2012; Fotso et al., 2008, 2009; Idowu et al., 2017; Kawakatsu et al., 2015; Kibiribiri et al., 2016; Kuwawenaruwa et al., 2016; Mustapha et al., 2018; Nwameme et al., 2014; Olusanya et al., 2010; Owiti et al., 2018; Rossier et al., 2014; Sasaki et al., 2010, 2011; Tann et al., 2007; Wakwoya et al., 2016; Westheimer et al., 2004; Woldeghebriel et al., 2017) and surveys (Adewuyi et al., 2017, 2018; Anastasi et al., 2017; Antai, 2010; Egondi et al., 2015; Machira & Palamulen, 2017; Mekonnen & Mekonnen, 2003; Ng’anjo Phiri et al., 2014; Ngandu et al., 2017; Rossier et al., 2014; Taffa & Chepngeno, 2005; Yaya et al., 2018). This review also included a few longitudinal studies (Bayu et al., 2015; Jennings et al., 2017; Mutua et al., 2011), cluster-randomized controlled trials (CRCT) (Ochola et al., 2013), RCT (Aidam et al., 2005b) and quasi-experimental studies (Kimani-Murage et al., 2016).

Studies included in this review targeted different population groups, mainly pregnant women (Aidam et al., 2005b; Bayu et al., 2015; Belayneh et al., 2014; Idowu et al., 2017; Kimani-Murage et al., 2016; Ngandu et al., 2017; Nwameme et al., 2014; Ochola et al., 2013; Owiti et al., 2018; Tann et al., 2007; Westheimer et al., 2004). These women represented reproductive age women (Adewuyi et al., 2017, 2018; Antai, 2010; Asundep et al., 2013; Babirye et al., 2014; Bayou et al., 2016; Demilew, 2017; Izugbara et al., 2009; Jennings et al., 2017; Kuwawenaruwa et al., 2016; Rossier et al., 2014; Woldeghebriel et al., 2017) including adolescent and young mothers (Mustapha et al., 2018) and those women who had other pregnancy outcomes (Fotso et al., 2008, 2009). Furthermore, reproductive age women with a history of at least one birth (Aidam et al., 2005a; Bellows et al., 2012; Kibiribiri et al., 2016; Machira & Palamulen, 2017; Yaya et al., 2018), a child(-ren) aged less than 5 years at the time of the survey (Abimbola et al., 2016; Mekonnen & Mekonnen, 2003; Ng’anjo Phiri et al., 2014; Sasaki et al., 2010, 2011) and under-5 children who completed vaccination (Kawakatsu et al., 2015) were included. Similarly, mothers attending vaccination clinics (Olusanya et al., 2010), children aged 12–23 months and women who gave birth (Egondi et al., 2015; Fatiregun & Okoro, 2012; Mutua et al., 2011), and mothers and their under-5 children with some childhood illnesses such as acute watery diarrhea (Adane et al., 2017) were represented in this review. Additionally, the study population for some of the studies included both males and females (Anastasi et al., 2017; Kimani-Murage et al., 2015), HIV positive adolescent women (Birungi et al., 2011), HIV positive mothers and their children (Wakwoya et al., 2016), commercial sex workers (CSWs) (Chege et al., 2002), refugee women (Kibiribiri et al., 2016), and internally migrated women (Mirkuzie, 2014). Qualitative studies enrolled key informants comprised of women who injected drugs (Ndimbii et al., 2018), care givers (Babirye et al., 2014), and women and men (Wilunda et al., 2016). Overall, 35 (66%) studies focused only on one or more maternal health services (ANC, PNC or skilled delivery) and 17 (32%) studies focused only on neonatal or child health services while 1 (2%) study addressed both maternal and child health services.

Sample size of the included studies ranged from a minimum of 110 participants (Kimani-Murage et al., 2015) to a maximum of 38,948 eligible women aged 15–49 years (Adewuyi et al., 2017). The included studies drew samples from different data sources including pregnancy history, clinical records (e.g., prevention of mother-to-child transmission of HIV (PMTCT)), multiple indicator cluster surveys, and population based national surveys (e.g., DHS). Qualitative studies used focus group discussions (FGDs), in-depth interviews (IDIs), and key-informant interviews (KIIs). Details on each study characteristics are described in Table 1.

**Table 1:** Summary characteristics of studies included in the systematic review

S.no.	Authors (year of publication)	Country	Study setting	Study design	Population	Sample size	Type of maternal health service/s assessed	Type of newborn and child health service/s assessed	Quality rating
1	Birungu et al. (2011)	Kenya	Urban, peri-urban	Cross-sectional analysis of pregnancy history data	HIV+ female adolescents; aged 15–19 years	393	Prenatal, skilled delivery, postnatal, Prevention of mother to child transmission of HIV (PMTCT)	Not applicable	Good
2	Taffa and Chepngeno (2005)	Kenya	Urban slums	Survey; the Nairobi Urban Demographic and Health Surveillance System (NUDSS)	Women and Children	3015	Not applicable	Health care for childhood illness	Good
3	Chege et al. (2002)	Kenya	Urban slums	Cross-sectional; Mixed methods	Commercial sex workers	385; 4 FGDs	Not applicable	Child health seeking; immunization	Fair
4	Tann et al. (2007)	Uganda	Urban	Cross-sectional retrospective community survey	Pregnant women	413	Antenatal care, skilled delivery	Not applicable	Good
5	Yaya et al. (2018)	Ethiopia	Urban, rural	Survey; 2011 demographic and health survey	Women aged between 15 and 49 years with a history of at least one birth	7540	Health facility delivery	Not applicable	Good
6	Ndimbii et al. (2018)	Kenya	Urban, peri-urban	Qualitative cross-sectional study	Women injecting drug users, together with key informants	45	Reproductive, maternal, neonatal and child health (RMNCH) services	Vaccination services	Good
7	Ngandu et al. (2017)	South Africa	Urban, peri-urban, rural	Cross-sectional survey of 2012 PMTCT program	Pregnant women	8618	PMTCT services, antenatal HIV care services	Not applicable	Good
8	Mustapha et al. (2018)	Uganda	urban	Cross-sectional, mixed methods	Postpartum mothers (HIV positive or negative)	418	PMTCT services	Not applicable	Fair
9	Anastasi et al. (2017)	Nigeria	Urban	Cross-sectional, community-based household survey	Males and females	Males: 598, Females: 3365	Perinatal health services	Not applicable	Fair
10	Adewuyi et al. (2017)	Nigeria	Rural, Urban	Cross-sectional survey	Women aged 15–49 years	38,948	Utilization of healthcare facility for childbirth	Not applicable	Good
11	Antai (2010)	Nigeria	Urban, rural, migrants	Nigeria demographic and health survey (2003 DHS)	Children and mothers aged 15–49 years	6029 children; 2735 mothers	Not applicable	Full immunization	Good
12	Mekonnen and Mekonnen (2003)	Ethiopia	Urban, rural	Ethiopia demographic and health survey (2000 DHS)	Women aged 15–49 years who had at least one child, aged < 5 years at the time of the survey	7987	Use of antenatal care services; Professional assisted delivery	Not applicable	Fair
13	Owiti et al. (2018)	Kenya	Urban	Cross-sectional	Pregnant women	396	Antenatal and postnatal services	Not applicable	Good
14	Adewuyi et al. (2018)	Nigeria	Urban, rural	Nigeria demographic and health survey (2013 DHS)	Urban and rural women	13,149 rural and 6503 urban	Antenatal care attendance/non-attendance	Not applicable	Good
15	Kawakatsu et al. (2015)	Kenya	Urban	Cross-sectional study	Children aged 12–59 months who have had completed full vaccination schedule	1902	Not applicable	Vaccination and child health services	Fair
16	Mutua et al. (2011)	Kenya	Urban	Longitudinal maternal and child health study	Women who gave birth and children aged 12–23 months who were expected to have received all the WHO-recommended vaccinations	1848	Not applicable	Vaccination services	Good
17	Machira and Palamulen (2017)	Malawi	Rural, urban	Malawi demographic and health survey (2010 DHS)	Urban, rural mothers	1454 urban and 12,322 rural	Postnatal care services	Not applicable	Good
18	Engodi et al. (2015)	Kenya	Urban slums	Nairobi cross-sectional slum survey	Women aged 15–49 years and children aged 12–23 months who were expected to have received all the recommended vaccinations	3892 women and 382 children		Full immunization status	Fair
19	Phiri et al. (2014)	Kenya	Rural, urban	Population-based survey	Women aged 15–49 years who have childbirth in previous 5 years	583 from Kenya	Facility delivery	Not applicable	Fair
		Tanzania				687 from Tanzania		Not applicable	
		Zambia				530 from Zambia		Not applicable	
20	Fotso et al. (2008)	Kenya	Urban slums	Prospective maternal health project carried out in 2006	Women who had a pregnancy outcome	1927	Frequency and timing of antenatal care	Not applicable	Good
21	Fotso et al. (2008)	Kenya	Urban slums	Maternal health project carried out in 2006	Women who had a pregnancy outcome	1927	Health facility delivery	Not applicable	Good
22	Bayu et al. (2015)	Ethiopia	Urban	Community-based follow-up study	Second- and third-trimester pregnant women who had planned for institutional delivery	522	Missed opportunities for institutional delivery	Not applicable	Fair
23	Kibiribiri et al. (2016)	South Africa	Urban, refugees	Cross-sectional, mixed methods study	Women whose children were aged 6 months or younger and who attended the immunization services	200	Prenatal care	Not applicable	Fair
24	Kimani-Murage et al. (2016)	Kenya	Urban slums	Quasi-experimental study	Pregnant women aged 12–49 and their respective babies (when born)	1110 mother–child pairs in the Intervention Study, 521 in the intervention arm and 581 in the control arm; and 487 mother–child pairs in the comparison study	Exclusive breastfeeding	Not applicable	Good
25	Kimani-Murage et al. (2014)	Kenya	Urban slums	Qualitative cross-sectional study	Women of reproductive age who were either pregnant, breastfeeding or had children under 5; community leaders, health care professionals; Community health workers; and traditional births attendants	110 participants (20 men 90 women)	Optimal breastfeeding practices	Not applicable	Good
26	Mirkuzie (2014)	Ethiopia	Urban, migrants	Qualitative cross-sectional study	Internal migrant women	45	Skilled care at birth	Not applicable	Good
27	Aidam et al. 2005a, 2005b)	Ghana	Urban	Cross-sectional	Women with infants 0–6 months, MCH clinics	376	Exclusive breastfeeding	Not applicable	Fair
28	Ochola et al. (2013)	Kenya	Urban slums	Cluster randomized controlled trial	HIV-negative women, 34–36 weeks pregnancy from an antenatal clinic	360	Exclusive breastfeeding	Not applicable	Good
29	Aidam et al. 2005a, 2005b)	Ghana	Urban	Randomized controlled trial	Pregnant women attending prenatal clinics	231	Exclusive breastfeeding	Not applicable	Fair
30	Bellows et al. (2012)	Kenya	Urban slums	Cross-sectional analysis of the 2004–2005 & 2006–2008 NUDHSS data	Females aged 12– 54 years and had either a live birth or stillbirth	1914 females aged 12–54 and 2448 females aged 12–54 years (and their children)	Facility based delivery, Skilled birth attendance	Not applicable	Fair
31	Olusanya et al. (2010)	Nigeria	Urban, inner city	Cross-sectional	Mothers and infants attending the BCG immunization clinics	6465 mothers (6558 infants)	Facility based delivery, Skilled birth attendance	Not applicable	Good
32	Izugbara et al. (2009)	Kenya	Urban, slums	Qualitative study	Purposefully selected women (16–64 years)	74	Facility based delivery	Not applicable	Fair
33	Rossier et al. (2014)	Kenya, Burkina Faso	Urban slums	Cross-sectional (analysis of 2009–2011 HDSS data)	Households living in several poor neighborhoods	3346 and 4239 births	Antenatal care attendance, place of delivery	Not applicable	Good
34	Belayneh et al. (2014)	Ethiopia	Urban	Cross-sectional study	Women who attended ANC at the University of Gondar Hospital	369	Early antenatal care booking	Not applicable	Fair
35	Babirye et al. (2014)	Uganda	Urban	Cross sectional, Mixed methods	Caregivers for quantitative and women and HCWs for the qualitative	Quantitative = 821 and Qualitative = 9 FGDs and 9 KIIs	Not applicable	Child health services (childhood immunization)	Good
36	Fatiregun and Okoro (2012)	Nigeria	Urban	Cross sectional study	Mother and their children aged 12–23 months	525	Not applicable	Child health services (childhood immunization)	Good
37	Idowu et al. (2017)	Nigeria	Urban	Cross sectional study	Participants with mean age of 30.3 ± 6.0 years	400	Antenatal care (utilization of skilled care among pregnant women)	Not applicable	Good
38	Demilew (2017)	Ethiopia	Urban	Cross sectional study	Mothers with mean age of 30 ± (4.3 SD) years	471	Not applicable	Infant and young child feeding practices	Good
39	Adane et al. (2017)	Ethiopia	Urban	Cross-sectional study	Mothers/caregivers and their corresponding under-5 children with acute diarrhea	472	Not applicable	Postnatal care (under-5 children with acute diarrhea)	Good
40	Abimbola et al. (2016)	Nigeria	Peri-urban	Cross-sectional study	Pregnant or have a baby in the last 5 years between the ages of 15–49 years	283	Antenatal care and delivery services		Fair
41	Wakwoya et al. (2016)	Ethiopia	Peri-urban	Cross sectional study	HIV positive mothers and their children with mean age of 29.9 years and 9.15 months, respectively	260	Not applicable	Child health services (infant feeding practices)	Good
42	Wilunda et al. (2016)	South Sudan	Peri-urban	Qualitative cross-sectional study	Women and men, and key informants	169 women and 45 men, and 18 key informant	Not applicable	Child health services	Good
43	Kuwawenaruwa et al. (2016)	Tanzania	Peri-urban	Mixed methods approach	Women	190	MCH care services	Not applicable	Fair
44	Woldeghebriel et al. (2017)	Ghana	Urban, refugees	Cross‐sectional study	Women	480	MCH care services	Not applicable	Good
45	Jennings et al. (2017)	Ghana, Nigeria	Peri-urban	Longitudinal study	Married or co-habiting women, aged 15–49 years	Ghana (n = 158) and Nigeria (n = 223)	Antenatal care and skilled birth Attendant	Not applicable	Good
46	Bayou et al. (2016)	Ethiopia	Urban	Cross-sectional study	Women aged 15–49 years	870	Antenatal care services	Not applicable	Good
47	Sasaki et al. (2010)	Zambia	Per-urban	Cross-sectional study	Caregivers with their corresponding under 5 year old children	1000	Not applicable	Child health services (danger signs in children)	Good
48	Westheimer et al. (2004)	Tanzania	Urban	Cross-sectional study	Women attending antenatal care services in and around Dar Es Salaam	10,991	MCH care services (Acceptance of HIV testing)	Not applicable	Good
49	Nwameme et al. (2014)	Ghana	Urban	Cross-sectional study	Women aged 17–46 years attending ante-natal care	390	MCH care services (compliance with obstetric referrals)	Not applicable	Fair
50	Asundep et al. (2013)	Ghana	Urban	Cross-sectional study	Women aged 19–48 years who presented for delivery at selected public hospitals and to traditional birth attendants	643	Antenatal care	Not applicable	Good
51	Sasaki et al. (2010)	Zambia	Peri-urban	Cross-sectional study	Children	268	Not applicable	Immunization	Good
52	Fotso et al. (2009)	Kenya	Urban poor setting	Cross-sectional study	Women who had a pregnancy outcome in 2004–2005	1927	Skilled delivery	Not applicable	Good
53	Govender et al. (2019)	South Africa	Urban	Cross-sectional study	Childbearing women	240	Antenatal care	Not applicable	Fair

### Quality of Included Studies

Most (31 out of 46) of the included observational quantitative studies were rated as good quality whereas the remaining 15 were rated as fair. The studies rated as good appropriately described the study design and methodologies, participant recruitment and inclusion, study population and description of assessment of exposure and outcomes. Four out of 5 qualitative studies included were rated good quality as they adopted appropriate methodologies, appropriate research designs, addressed ethical issues, and sufficiently provided evidence of rigorous analysis. Details of the quality assessment of these studies are shown in Supplementary 2 (Tables 1 and 2). One out of 2 RCTs included had an overall low risk bias whereas the other had some concerns related to measurement of the outcome and selection of the reported results as shown in Supplementary 2 (Table 3).

## Maternal, Newborn and Child Health Services

The main outcomes measured included utilization of maternal health care services (Adewuyi et al., 2018; Anastasi et al., 2017; Birungi et al., 2011; Fotso et al., 2008; Kuwawenaruwa et al., 2016; Machira & Palamulen, 2017; Mustapha et al., 2018; Ndimbii et al., 2018; Ng’anjo Phiri et al., 2014; Ngandu et al., 2017; Nwameme et al., 2014; Owiti et al., 2018; Westheimer et al., 2004; Woldeghebriel et al., 2017) including frequency and/or timing of ANC services (Abimbola et al., 2016; Asundep et al., 2013; Bayou et al., 2016; Belayneh et al., 2014; Fotso et al., 2008; Idowu et al., 2017; Jennings et al., 2017; Kibiribiri et al., 2016; Rossier et al., 2014; Tann et al., 2007), facility-based/institutional delivery and/or skilled birth attendance (Abimbola et al., 2016; Bayu et al., 2015; Bellows et al., 2012; Fotso et al., 2009; Idowu et al., 2017; Izugbara et al., 2009; Mirkuzie, 2014; Ng’anjo Phiri et al., 2014; Olusanya et al., 2010; Rossier et al., 2014), and postnatal care (PNC) services (Adane et al., 2017; Demilew, 2017; Kawakatsu et al., 2015; Machira & Palamulen, 2017; Mutua et al., 2011). Additionally, exclusive breast feeding, optimal breastfeeding and other child health services were variously assessed (Aidam et al., 2005a, 2005b; Antai, 2010; Chege et al., 2002; Egondi et al., 2015; Fatiregun & Okoro, 2012; Kimani-Murage et al., 2015, 2016; Ochola et al., 2013; Sasaki et al., 2010, 2011; Wakwoya et al., 2016; Wilunda et al., 2016). Some studies also assessed factors associated with non-utilization of MNCH care services (Adewuyi et al., 2017; Mekonnen & Mekonnen, 2003; Taffa & Chepngeno, 2005; Yaya et al., 2018) (Table 2).

**Table 2:** Summary of findings

Authors (year of publication)	Factors identified as contributors to inequalities in access to and utilization of maternal, newborn and child health (MNCH) services in SSA			
	Antenatal care (ANC)	Postnatal care (PNC)	Skilled delivery	Newborn and child health services
Birungi et al. (2011), Kenya	*PMTCT* Pregnancy order: OR, 95% CI 0.5, 0.3–0.9 Husband support, 4.9, 1.8–13.1	Not applicable	Pregnancy order: OR, 95% CI 0.6, 0.4–0.9	Not applicable
Taffa and Chepngeno (2005), Kenya	Not applicable	Not applicable	Not applicable	Lack of money (HH expenditure < 7431 Ksh): OR, 95% CI 2.2, 1.57–3.0
Chege et al. (2002), Kenya	Not applicable	Not applicable	Not applicable	Reasons for non-immunization: lack of proper instruction by clinic staff, 0.8%; forgetting 0.8%; pressure of work 1.6%. Reasons for failure to seek treatment: lack of finance 71.4% and pressure of work: 28.6%. Focus group discussion: Use of intoxicants found to be an obstacle to health seeking for children
Tann et al. (2007), Uganda	Not applicable	Crowding at home: < 3 years > 3 in the household: OR, 95% CI 1.96, 1.19–3.20	Lower education: OR, 95% CI 3.07, 1.49–6.31; Lack of electricity: 3.47, 1.51–7.97; Crowded household: 2.71, 1.42–5.16	Not applicable
Yaya et al. (2018), Ethiopia	Not applicable	Not applicable	Education (primary versus none): OR, 95% CI 3.72, 2.50–5.54); Sec/higher 1.75, 1.30–2.35 Richest versus poorest: 4.97, 2.41–10.25 Age at first birth 18/18+ years < 18: 1.92, 1.44–2.55	Not applicable
Ndimbii et al. (2018), Kenya	Stigmatizing attitudes from health care workers, long waiting times		Not applicable	Lack of a holistic view of children health needs
Ngandu et al. (2017), South Africa	*PMTCT* The largest contributions to the observed inequality between low and high wealth groups were from: inequalities in province (contribution, 65.27%), age (44.38%), wealth group (24.73%) and transport means (21.61%)	Not applicable	Not applicable	Not applicable
Mustapha et al. (2018), Uganda	*PMTCT* HIV+ mothers: OR, 95%CI 18.2 (95% CI 9.0–36.7); Mothers aged 20–24 years 15–19 years: 1.9, 1.1–3.6; Stigma, financial constraints, non-disclosure, and lack of partner and family support were key demotivating factors	Not applicable	Not applicable	Not applicable
Anastasi et al. (2017), Nigeria	Stigma from healthcare Long waiting times		Not applicable	Not applicable
Adewuyi et al. (2017), Nigeria	Not applicable	Not applicable	*Home delivery* Age ≥ 36 years vs 20-35 years: OR, 95% CI 0.77, 0.63–0.95; Education None vs secondary/higher: 1.52, 1.12, 1.94 Birth order: 1 vs ≥ 4: 0.58, 0.44–0.76	Not applicable
Antai (2010), Nigeria	Not applicable	Not applicable	Not applicable	Full immunization: Urban migrant vs non-migrant: OR, 95%CI 1.54, 1.11–2.15 Differences in full immunization between the migrant and non-migrant groups partly explained by unequal utilization of health care services
Mekonnen and Mekonnen (2003), Ethiopia	Primary vs no educ. OR, 95% CI 1.7, 1.0–3.0; Sec vs no educ. 3.8, 2.1–6.9 Married 2.4 (1.3–4.1)	Not applicable	Primary versus no education: OR, 95% CI 2.3, 1.3–4.1; Sec vs no educ. 7.2, 4.1–12.5	Not applicable
Owiti et al. (2018), Kenya		Not applicable	Positive perception of the public health facility within closest proximity: OR, 95% CI 17.3, 4.5–66.6 Travelling by foot (ref = private car): 0.075, 0.019–0.293 Attending ANC at a private health facility (0.049, 0.012–0.196) and an NGO health facility (0.081, 0.028–0.235)	Not applicable
Adewuyi et al. (2018), Nigeria	*ANC underuse* Lack of maternal education: AOR, 95% CI 1.44–1.10, 1.87 Poor HHs: 2.05, 1.51–2.79 Lack of husband’s education: 2.16, 1.68–2.75 Mothers age (ref = 35 years) < 20 years: 1.75, 1.13–2.70 & 20–34 years 1.25, 1.03–1.49) No access to health insurance 3.41, 1.53, 7.58	Not applicable	Not applicable	Not applicable
Kawakatsu et al. (2015), Kenya	Not applicable	Not applicable	Not applicable	*Full vaccination* Highest wealth quintile: AOR, 95% CI 2.49, 1.33–4.64 Community with high media coverage devices 1.50, 1.029–2.198 Participation in the mass immunization campaigns: 1.63, 1.15–2.30
Mutua et al. (2011), Kenya	Not applicable	Not applicable	Not applicable	*Full coverage* Primary education, OR, 95%CI 1.30, 1.01–1.67) Mother age (ref < 20) 20-24 years: 1.48,1.06–2.08) & 25-29 years: 1.76, 1.18–2.62 Parity (ref = 1), parity 2: 0.66, 0.5–0.87; parity 3: 0.56, 0.41–0.78) Poverty
Machira and Palamulen (2017), Malawi	Not applicable	Access to care/services	Not applicable	Not applicable
Engodi et al. (2015), Kenya	Not applicable	Not applicable	Not applicable	*Contributor to overall inequality in immunization coverage across wealth quintiles* Mother’s level of education Birth order Involvement in any income generating activity
Phiri et al. (2014), Kenya	Not applicable	Not applicable	SES (low to high): OR, 95% CI 1.14, 1.09–1.16 ANC visits (0–9): 1.35, 1.00–1.81	Not applicable
Tanzania	Not applicable	Not applicable	Trust quality (1–4): 1.63, 1.21–2.19 Perceived cost of care: 1.65, 1.15–2.37	Not applicable
Zambia	Not applicable	Not applicable	High SES: 1.11.04–1.17 Single/divorced vs married 0.43, 0.21–0.88—perceived cost (not at all to very much (1–5): 1.31, 1.02–1.77 Perceived distance (low to high): 0.77, 0.66–0.90	Not applicable
Fotso et al. (2008), Kenya	Not applicable	Not applicable	The proportion of non-health facility deliveries steadily declined with education and wealth and increased with parity	Not applicable
Fotso et al. (2008), Kenya	Not applicable	Not applicable	Secondary+ vs primary: OR, 95% CI 1.611, 1.29–2.02 Working vs not working: 1.31, 1.05, 1.63 HH wealth (ref = poorest): middle 1.26, 1.00–1.59 & least poor: 2.11, 1.63–2.75	Not applicable
Bayu et al. (2015), Ethiopia	Not applicable	Not applicable	*Unplanned home delivery* Single women: AOR, 95% = 2.34, 1.17–4.68 Illiterate mothers: 6.14, 2.20–17.2 Absence of antenatal clinic visit for indexed pregnancy: 3.11, 1.72–5.61 Poor autonomy: 2.11, 1.27–3.49 Absence of birth preparedness and complication readiness: 3.83, 2.19–6.70	Not applicable
Kibiribiri et al. (2016), South Africa	Refugees dislike facility due to health care workers’ xenophobia (43.6%), carelessness (42.3%), and inability to communicate with refugees (37.2%) Higher proportion of refugees did not receive enough information about labor and child birth (39.2% vs 24.4%), self-health care during pregnancy (44.6% vs 26.1%)	Not applicable	Not applicable	Not applicable
Kimani-Murage et al. (2016), Kenya	Not applicable	Not applicable	Not applicable	AOR, 95% CI for EBF from birth to 6 months: 66.9, 45.4–96.4; 84.3, 40.7–174.6; and 3.9 (95% CI 1.8–8.4) for the MIYCN-intervention, MIYCN-control and comparison group, respectively, compared with the pre-intervention group
Kimani-Murage et al. (2014), Kenya	Not applicable	Not applicable	Not applicable	*Optimal breastfeeding* Poverty, livelihood and living conditions (work e.g. commercial sex work, food insecurity, living arrangements, alcoholism—early and single motherhood Poor social and professional support Poor knowledge, myths and misconceptions HIV (fear of MCT, stigma) Unintended pregnancies
Mirkuzie (2014), Ethiopia	Not applicable	Not applicable	Social influences, Physical access to health facility Risk perceptions Perceived quality of care and disrespectful care	Not applicable
Aidam et al. 2005a, 2005b), Ghana	Not applicable	Not applicable	Not applicable	*Exclusive breastfeeding since birth* Delivery at a hospital/polyclinic: R, 95% CI 1.96, 1.08–3.54) High socioeconomic status (women living in their own houses compared to those who rent): 3.96, 1.02–15.49
Ochola et al. (2013), Kenya	Not applicable	Not applicable	Not applicable	*Exclusive breastfeeding* Intervention versus control: ARR, 95% CI 4.01, 2.30–7.01
Aidam et al. (2005a, 2005b), Ghana	Not applicable	Not applicable	Not applicable	The percentage of exclusive breastfeeding during the 6 month significantly higher among Intervention Group1 and Intervention Group 2 (39.5%) than among control group (19.6%)
Bellows et al. (2012), Kenya	*Any ANC visit* Purchase of voucher: OR, 95% CI 11.4, 4.3–42.9	Not applicable	*Facility based delivery* Age 23–34 versus 12–23: OR, 95% CI 1.2, 1.04–1.47 Secondary education: 1.6, 1.28–1.98 Least poor 1.3, 1.15–1.53 Facility based delivery (12.9, 8.9–19.3) & skilled birth delivery (1.2, 1.1–1.4) increased during the voucher programme period	Not applicable
Olusanya et al. (2010), Nigeria	Not applicable	Not applicable	*Delivery outside hospital* Teenage mothers: OR, 95% CI 1.63, 1.12–2.37 No education: 3.45, 2.18–5.45 Primary education: 2.19, 2.13–3.66 Secondary education: 1.71, 1.37–2.14 Unemployed: 1.31, 1.05–1.63 Belonging to low social class: 1.51, 1.02–2.44 or middle 1.61, 1.12–2.33 Living in accommodation with shared sanitation facilities: 2.031.67–2.48 Being multiparous: 1.58, 1.27–1.97 *Lack of skilled birth delivery* Belonging to low (2.31; 1.07–4.97), or middle (2.53, 1.21–5.27) social class	Not applicable
Izugbara et al. (2009), Kenya		Not applicable	*Barriers to hospital based delivery* Very exorbitant and often out of their reach Hospital-based providers characterized as harsh and uncaring, Physical proximity of services Fear of HIV testing and counselling	Not applicable
Rossier et al. (2014), Kenya	*ANC use at least 1 visit* Women with the least education: OR, 95% CI 0.36, 0.15–0.87; Not poor: 2.35, 1.39–3.96 Living in the wealthier area 0.56 0.321–0.989 Increase parity decreased ANC visit	Not applicable	Skilled delivery increased with Increased education Antenatal care attendance	Not applicable
Burkina Faso	*ANC at least 4*+ *visit* Not poor 1.54, 1.22–1.94; Secondary education 1.59, 1.21–2.10	Not applicable	Secondary education increased skilled birth delivery	Not applicable
Belayneh et al. (2014), Ethiopia	*Early ANC visit* Younger age: AOR, 95% CI 3.83, 1.8 9–10.53 Formal education: 1.06, 1.03–7.61 Early ANC visit: 2.39, 2.23–9.86	Not applicable	Not applicable	Not applicable
Babirye et al. (2014), Uganda	Not applicable	Not applicable	Not applicable	*Low utilization of immunization services* Lack of financial support for immunization activities—intermittent availability of vaccines and transport for immunization services at both private and public facilities
Fatiregun and Okoro (2012), Nigeria	Not applicable	Not applicable	Not applicable	*Complete immunization status* Maternal age < 30 years: AOR, 95% CI 2.26, 1.27–4.03 Availability of an immunization card at first contact: 7.724.43–13.44 < 3 children: 2.22, 11.1–4.42 Completion of post-secondary education: 2.34,1.12–4.47 Maternal unemployment 1.71, 1.01–2.89
Idowu et al. (2017), Nigeria	Not applicable	Not applicable	*Skilled birth attendance* Maternal age ≤ 19 years: AOR, 95% CI 0.03, 0.003–0.25 Higher education: 10.94, 3.60–33.26 Having only one child: 4.33, 1.18–15.82 Having ≤ 4 ANC attendance Residing near delivery sites	Not applicable
Demilew (2017), Ethiopia, Ethiopia	Not applicable	Not applicable	Not applicable	*Knowledge on infant feeding* Education beyond primary education: AOR, 95% CI 2.5, 1.5–3.9 Possession of radio 1.7, 1.1–2.7 Antenatal care attendance: 2.4, 1.5–4.0 Having an employed husband: 2.3, 1.2, 4.4
Adane et al. (2017), Ethiopia	Not applicable	Not applicable	Not applicable	*Health seeking behaviors for under-5 children with acute diarrhea* Literacy of mother: OR, 95%CI 2.4, 1.4–4.1 Occupation of mothers/caregiver: 2.6, 5–4.6 Household monthly income ≥ 50 US$: 2.9; 1.5–5.6 Availability of nearest health facilities within 15 min walking distance: 3.3, 1.7–6.6
Abimbola et al. (2016), Nigeria	*Barriers to the utilization of ANC* Lack of money *Enhancers of utilization* level of education, employment status and higher parity	Not applicable	Not applicable	Not applicable
Wakwoya et al. (2016), Ethiopia		Not applicable	Not applicable	*Infant feeding* Higher education: AOR, 95% CI 5.3, 1.25–22.1 Antenatal care attendance: 5.5, 1.5–20.16 On anti-retro viral therapy (ART): 6.5, 1.88–22.51 Disclosed HIV status: 7.1, 1.26–39.76)
Wilunda et al. (2016), South Sudan	Not applicable	Not applicable	*Barriers to institutional childbirth* Access and lack of resources e.g. long distance to health facilities, lack of transportation means, referral problems Socio-cultural context and conflict: e.g. insecurity, influence of the husband, lack of birth preparedness Perceptions about pregnancy and childbirth e.g. perceived benefit of institutional childbirth Perceptions about the quality of care	Not applicable
Kuwawenaruwa et al. (2016), Tanzania	Not applicable	Not applicable	*Decision about where to deliver* Individual factors e.g. complications during previous pregnancy, male involvement in decision-making Financial factors e.g. cost of transportation, other costs Health system factors e.g. providers and client relationship	Not applicable
Woldeghebriel et al. (2017), Ghana	Not applicable	Not applicable	Not applicable	*Early breastfeeding for* ≥ *6 months* Borrowing money from a neighbor or family member: OR, 95% CI 1.53, 1.05, 2.23 Liberian refugees living in Ghana for 8 years or more (ref: Ghanaians): 1.78, 1.02, 3.09
Jennings et al. (2017), Ghana & Nigeria	Not applicable	Not applicable	*Skilled delivery* Women in household with savings: AOR, 95% CI 2.81, 1.25–6.33 > 3 positive economic characteristics 2.69, 1.21–5.99	Not applicable
Bayou et al. (2016), Ethiopia	*Adequate ANC visit/care* Higher education: OR, 95% CI 2.69, 1.29–5.63 Never-married/formerly married women: 0.38, 0.20–0.73 ANC follow ups in private facilities 2.16, 1.02–4.49	Not applicable	Not applicable	Not applicable
Sasaki et al. (2010), Zambia		N/A	N/A	*Care seeking for danger signs in children-baseline* Lower-income OR, 95% CI = 0.47, 0.25–087 farthest distance 0.30, 0.13–0.66 *3 years after the intervention* Frequent attendance at growth monitoring sessions (ref:4–6 times): –No attendance 0.31, 0.15–0.65 –1–3 attendance 0.43, 0.22–0.88
Westheimer et al. (2004), Tanzania	*PMTCT-odds of accepting HIV testing* Aged 20–24 years: OR, 95%CI 0.90, 0.78–1.03 Unknown spouse’s occupation: 1.41, 1.15–1.71 Cohabiting with the partner: 1.14, 1.03–1.26 Higher education: 0.79, 0.64, 0.96 3 children living at home: 0.83, 0.72, 0.97	Not applicable	Not applicable	Not applicable
Nwameme et al. (2014), Ghana	*Noncompliance with maternal referrals* Major causes include: financial problems (46.2%), attitude of nurses at the referral centers (10.8%), fear of surgery (7.7%) and distance to referral centers (4.6%)	Not applicable	Not applicable	Not applicable
Asundep et al. (2013), Ghana	*ANC attendance influenced by* Cost: AOR, 95% CI 1.86, 1.04–3.32 Distance to health facility: 2.24, 1.00–5.03 Cultural beliefs: 2.59, 0.95–7.08	Not applicable	Not applicable	Not applicable
Sasaki et al. (2010), Zambia	Not applicable	Not applicable	Not applicable	*Access to immunization* Longer distances to a service point → immunization coverage of DPT3: OR, 95% CI 0.24, 0.10–0.56 and measles 0.38, 0.17–0.82 Female headed household: 0.42, 0.19–0.95 After intervention, distance and HH head were no more associated with immunization overage
Fotso et al. (2009), Kenya	Not applicable	Not applicable	*Deliver in appropriate health facilities*—among middle and least poor households, high overall autonomy women were slightly more likely to deliver in appropriate health facilities Delivery at equipped health facility associated with: increased education, ANC counseling, wanted pregnancies. Age < 25 years & increase parity	Not applicable
Govender et al. (2019), South Africa	*Use of ANC influenced by* Emotional vulnerability, i.e. fear, loneliness, shame and disgrace Financial barriers Attitude of health care workers, long queues at health facilities, and long distance to health facilities Level of education	Not applicable	Not applicable	Not applicable

### Antenatal Care Services

Twelve studies assessed the utilization, frequency and/or timing of ANC (Abimbola et al., 2016; Adewuyi et al., 2018; Asundep et al., 2013; Bayou et al., 2016; Belayneh et al., 2014; Birungi et al., 2011; Fotso et al., 2008; Kibiribiri et al., 2016; Mekonnen & Mekonnen, 2003; Ndimbii et al., 2018; Rossier et al., 2014; Tann et al., 2007). The level of ANC services utilization ranged from 22% in Burkina Faso (Rossier et al., 2014) to 96% in Uganda (Tann et al., 2007). Timely ANC booking was reported by 47.4% pregnant women (Belayneh et al., 2014). Despite high ANC coverage (83.1%) (Mekonnen & Mekonnen, 2003), median for 4+ ANC visit was 22% and only 1 in 10 (11%) women received adequate ANC services in Ethiopia (Bayou et al., 2016), with 43% underutilization of the recommended services in South Africa (Govender et al., 2019). There was a 78.4% HIV test acceptance rate among women who were attending ANC in Tanzania (Westheimer et al., 2004).

Variations in access and utilization of ANC services were found by factors such as younger maternal age, religion, marital status, education, employment status and wealth. Women aged less than 20 or between 20 and 34 years were up to 1.75 times more likely to under use ANC services when compared to mothers aged 35 years or older in Nigeria (Adewuyi et al., 2018). However, younger maternal age was also associated with a higher odds of early ANC booking in in Ethiopia (Belayneh et al., 2014). Married women had higher odds of utilizing ANC services when compared to never-married or those cohabiting/living together (Bayou et al., 2016). For instance, ANC service use was double among women who were married when compared to unmarried women in Ethiopia (Mekonnen & Mekonnen, 2003). The frequency and timing of ANC visit was improved with an increase in women’s educational status (Abimbola et al., 2016; Adewuyi et al., 2018; Bayou et al., 2016; Fotso et al., 2008; Mekonnen & Mekonnen, 2003; Rossier et al., 2014), education of the husband (Adewuyi et al., 2018), and wealth status (Abimbola et al., 2016; Adewuyi et al., 2018; Fotso et al., 2008; Rossier et al., 2014). Women who possessed formal education were more likely to make early ANC bookings (AOR 1.1) (Belayneh et al., 2014). The uptake of ANC services was also found to be improved by employment status (Abimbola et al., 2016).

Variations were also found by the type of health facilities women have access to and discriminating attitudes by health personnel. Pregnant women attending private health clinics were more likely to receive adequate ANC services than those women who had ANC follow-ups in public health facilities among urban slum residents in Ethiopia (Bayou et al., 2016). Stigmatizing attitude from health care workers was found to be one of the reasons for low utilization of ANC among women living in the slums of Nairobi (Ndimbii et al., 2018). In Nigeria, the disparity in the quality of prenatal care received by pregnant refugee women and “locals” was also high-characterized by inadequate provision of relevant ANC information and restriction to access of these services (Kibiribiri et al., 2016). This finding was complimented by language barrier and perceived discrimination (Kibiribiri et al., 2016). Notably, women who had no access to health insurance in South Africa had three times as high odds of ANC under use relative to women who had health insurance (Adewuyi et al., 2018).

### Facility-Based and Skilled Delivery

Nineteen studies examined skilled birth attendance (SBA) (Abimbola et al., 2016; Adewuyi et al., 2017; Bayu et al., 2015; Bellows et al., 2012; Birungi et al., 2011; Fotso et al., 2009; Idowu et al., 2017; Izugbara et al., 2009; Jennings et al., 2017; Kuwawenaruwa et al., 2016; Mekonnen & Mekonnen, 2003; Ndimbii et al., 2018; Ng’anjo Phiri et al., 2014; Olusanya et al., 2010; Owiti et al., 2018; Rossier et al., 2014; Tann et al., 2007; Wilunda et al., 2016; Yaya et al., 2018). In these studies, the proportion of health facility/institutional deliveries ranged from as low as 18.2% in Nigeria (Olusanya et al., 2010) to as high as 97% in Kenya (Owiti et al., 2018). Higher rates of facility delivery were also reported in Burkina Faso (95%) (Rossier et al., 2014), Ghana (89%) (Jennings et al., 2017), Zambia (77.1%) (Ng’anjo Phiri et al., 2014), Tanzania (74.8%) (Ng’anjo Phiri et al., 2014), Ethiopia (71.2%) (Bayu et al., 2015), Nigeria (63%) (Jennings et al., 2017) and Uganda (63%) (Tann et al., 2007).

Variations in access and utilization of facility/skilled delivery services were found by factors such as younger maternal age, marital status, education, employment status, occupational status of the husband, and wealth status. Women aged 18 years old at first childbirth had significantly less access to facility-based delivery services compared to women aged less than 18 years old (OR 1.92) (Yaya et al., 2018). A study conducted in Nigeria also showed that younger mothers (teenage women) were 1.63 times more likely to deliver outside hospital facilities (Olusanya et al., 2010), and were less likely to use SBA (Idowu et al., 2017). Married women were also more likely deliver in health facility than single or divorced women in Kenya (Ng’anjo Phiri et al., 2014) and in Ethiopia (Bayu et al., 2015). There was an increased utilization of SBA both among married and single women who were educated (Adewuyi et al., 2017; Bayu et al., 2015; Bellows et al., 2012; Fotso et al., 2008; Idowu et al., 2017; Kuwawenaruwa et al., 2016; Mekonnen & Mekonnen, 2003; Olusanya et al., 2010; Tann et al., 2007; Yaya et al., 2018). Particularly, secondary education was an important factor that increased facility delivery/use of SBA services in Kenya (Bellows et al., 2012; Fotso et al., 2008; Rossier et al., 2014). In addition to low level of maternal education, low level of paternal education was also associated with an increased likelihood of home delivery in Nigeria (Adewuyi et al., 2017). Employed and working women deliver in health facilities and use SBA (Fotso et al., 2008; Olusanya et al., 2010).

Occupational status of the husband was also found to be significantly associated with the place of delivery in Nigeria (Abimbola et al., 2016). Wealthier women were significantly more likely to use health facility delivery and SBA (Abimbola et al., 2016; Adewuyi et al., 2017; Fotso et al., 2008; Izugbara et al., 2009; Jennings et al., 2017; Kuwawenaruwa et al., 2016; Rossier et al., 2014; Tann et al., 2007; Yaya et al., 2018). Women in the richest wealth quantile were five times more likely to use delivery services by skilled personnel in Ethiopia (Yaya et al., 2018), those in the least poor wealth quantile were 2.1 times more likely to use facility delivery with skilled attendant while those from the middle wealth quantile were 1.3 times compared to women in the poorest wealth quantile to use facility delivery with skilled attendant (Fotso et al., 2008). Poorer women were more likely to deliver at home, or to use TBAs or untrained assistants at birth (Adewuyi et al., 2017; Rossier et al., 2014; Tann et al., 2007). In the same vein, financial factors such as costs related to transportation and delivery services, and poor road network were barriers to facility delivery in Kenya, South Sudan, Tanzania, and Uganda (Kuwawenaruwa et al., 2016; Ng’anjo Phiri et al., 2014; Tann et al., 2007; Wilunda et al., 2016).

Variations in access and utilization of facility delivery services were also found by ability to pay for services and proximity of health facilities. Service payments in health facilities were also mentioned as the main barriers to institutional childbirth (Wilunda et al., 2016). Close proximity to health facilities promoted an increase in facility-based deliveries (Idowu et al., 2017; Izugbara et al., 2009; Ng’anjo Phiri et al., 2014; Owiti et al., 2018; Rossier et al., 2014; Wilunda et al., 2016) and the likelihood of SBA (AOR 11.5) (Idowu et al., 2017). Conversely, living far from health facilities reduced deliveries in health facilities (Ng’anjo Phiri et al., 2014; Rossier et al., 2014). Among women living in areas with high HIV related stigma like Nairobi slums, fear of HIV testing and counselling service was a significant barrier to a facility delivery (Izugbara et al., 2009).

### Utilization of Postnatal Care Services

Postnatal care services mainly included HIV testing (Ngandu et al., 2017) and vaccination (Mutua et al., 2011; Ndimbii et al., 2018) thereby six of the included studies examined these services (Birungi et al., 2011; Machira & Palamulen, 2017; Mirkuzie, 2014; Mutua et al., 2011; Ndimbii et al., 2018; Ngandu et al., 2017). The level of PNC services use ranged from 30.1% in Uganda (Mustapha et al., 2018) to 94.5% in Kenya (Mutua et al., 2011). For example, in Uganda, the uptake of PMTCT service utilization among adolescent and young postpartum mothers was 30.1% in Uganda (Mustapha et al., 2018).

Variations in access and utilization of PNC were found by wealth status and exposure to mass media. For instance, post-natal HIV testing was better utilized in the lower 40% wealth group than the higher wealth group in South Africa (Ngandu et al., 2017). Knowledge on pregnancy complications was also associated with an increased likelihood of PNC services use (AOR 1.6) (Machira & Palamulen, 2017). Women who were exposed to mass media (radio or television) significantly improved the use of PNC services (AOR 1.4) (Machira & Palamulen, 2017).

At health facility level, stigmatizing attitudes from health care workers were the main reasons for low utilization of available services from postnatal clinics (Ndimbii et al., 2018). Physical barriers to access PNC services including means of transportation and distance to health facilities were other reasons to underutilization of PNC services (e.g., HIV testing) (Machira & Palamulen, 2017; Ngandu et al., 2017).

### Child Health Services

Child health services that were examined include exclusive breastfeeding (EBF) practice, vaccination coverage (Antai, 2010; Babirye et al., 2014; Chege et al., 2002; Egondi et al., 2015; Fatiregun & Okoro, 2012; Kawakatsu et al., 2015; Mutua et al., 2011; Sasaki et al., 2011) and health care for childhood illnesses (Taffa & Chepngeno, 2005) including care for under 5 children with acute diarrhea (Adane et al., 2017) and treatment of danger signs in children (Sasaki et al., 2010). The percentage of EBF ranged from 51.6% in Ghana (Aidam et al., 2005a) to 85.8% among HIV positive mothers in Ethiopia (Wakwoya et al., 2016). Although a large percentage (99.7%) of mothers were breastfeeding and 85.6% of these mothers had planned EBF during delivery, the proportion of EBF was only 51.6% in Ghana (Aidam et al., 2005a). A wide difference also existed in vaccination coverage. The rate of full vaccination ranged from as low as 8.5% among urban migrant women in Nigeria (Antai, 2010) to 96% in disadvantaged women in Kenya (Chege et al., 2002). The percentage of mothers/caregivers who sought care for under 5 children with acute diarrhea was 70.8%, either at home (14.2%) or health facilities (56.6%), although 29.2% caregivers reported that they did not seek any care (Adane et al., 2017). Similarly, 60.5% women sought care outside home for childhood illnesses in Nairobi slums (Taffa & Chepngeno, 2005).

Variations in access and utilization of child health services were found by such factors as maternal age, maternal education, marital and employment status of the mother. Early and single motherhood was related with poor breastfeeding practices in Kenya (Kimani-Murage et al., 2015). As compared to younger mothers (< 20 years), older mothers (> 30 years) were more likely to fully vaccinate their children (Fatiregun & Okoro, 2012; Mutua et al., 2011). Maternal education played a significant role for improved child health seeking behavior and adherence to the recommended child health services (Adane et al., 2017; Egondi et al., 2015; Fatiregun & Okoro, 2012; Mutua et al., 2011; Wakwoya et al., 2016). More than two-thirds (78%) disparity for vaccination was explained by mothers’ educational status (Egondi et al., 2015). Mothers who completed primary school education (AOR 1.3) and post-secondary education (AOR 2.3) were more likely to fully vaccinate their children (Fatiregun & Okoro, 2012; Mutua et al., 2011) compared to mothers without any education. Similarly, higher educational attainment increased the level of EBF (Aidam et al., 2005a). Indeed, literacy of mothers/caregivers was significantly associated with better health-seeking behavior for acute diarrhea (AOR 2.4) in Ethiopia (Adane et al., 2017).

Maternal occupation and conditions of work also affected seeking for and utilization of child health services (Chege et al., 2002; Fatiregun & Okoro, 2012; Kimani-Murage et al., 2015). Poverty was also one of the main factor for poor health seeking behavior for common childhood illnesses and less adherence to recommended child health services (Adane et al., 2017; Babirye et al., 2014; Chege et al., 2002; Egondi et al., 2015; Kawakatsu et al., 2015; Mutua et al., 2011; Sasaki et al., 2010; Taffa & Chepngeno, 2005; Woldeghebriel et al., 2017). Poverty reduced the odds of full vaccination coverage (Mutua et al., 2011), where inequality in vaccination was more concentrated among children from poorer families in Kenya (Egondi et al., 2015). Women who were living in their own homes were also more likely to exclusively breastfeed compared to those who lived in rental houses (AOR 4) (Aidam et al., 2005a). Similarly, availability of financial support borrowing money from a neighbor or family member positively influenced EBF practice (AOR 1.5) (Woldeghebriel et al., 2017). Maternal unemployment was associated with increased odds of complete child vaccination (AOR 1.7) in Nigeria (Fatiregun & Okoro, 2012).

At health facility level, variations in utilization of child services were linked to proximity and quality of services. The proximity of health facilities (within 15 min walking distance) significantly increased health-seeking behavior of women for under-5 children with acute diarrhea (AOR 3.3) (Adane et al., 2017). Inadequate human resources and shortage of vaccines or other supplies were the main barriers for childhood vaccination in Nigeria (Babirye et al., 2014). Children who stayed in a community with high coverage of media (AOR 1.5) and participated in mass vaccination campaigns (AOR 1.6) had full vaccination coverage in Kenya (Kawakatsu et al., 2015). Home-based intensive sessions were also effective in promoting EBF among children in low socio-economic conditions in Kenya (Ochola et al., 2013). Poor social and professional support, poor knowledge, myths and misconceptions in families and communities were related to poor breastfeeding practices in the country (Kimani-Murage et al., 2015), but having a positive attitude to EBF was associated with increased EBF practice in Ghana (Aidam et al., 2005a).

### HIV Testing and Prevention of Mother-to-Child Transmission of HIV

Levels of HIV/PMTCT services uptake were found to vary significantly by certain sociodemographic characteristics (Birungi et al., 2011; Mustapha et al., 2018; Ngandu et al., 2017; Westheimer et al., 2004) including age, education, male involvement and financial status. One in three mothers aged 20–24 years had utilized PMTCT services compared to 20% of younger mothers aged 15–19 years old (AOR 1.9) (Mustapha et al., 2018). Women with higher education, compared to those with low levels of education, were less likely to accept HIV screening test (AOR 0.8) in Tanzania (Westheimer et al., 2004). However, women whose spouses’ occupation were unknown were more likely to accept HIV screening test (AOR 1.4) (Westheimer et al., 2004). Male involvement during pregnancy was also associated with PMTCT services utilization. For instance, women who received support from their husbands during pregnancy were more likely to receive PMTCT services (AOR 4.9) (Birungi et al., 2011). Additionally, financial constraints were mentioned as the main reasons for poor PMTCT uptake in Uganda (Mustapha et al., 2018). Early HIV testing was better in lower wealth index as compared to the highest wealth index in South Africa (Ngandu et al., 2017) where access to means of transportation also influenced early HIV testing (Ngandu et al., 2017).

## Discussion

This systematic review was conducted to assess the coverage and inequalities in access to and use of MNCH services in the SSA region with a specific focus on urban settings. Ensuring MNCH services utilization throughout pregnancy, childbirth and postnatal period is a key in improving pregnancy and child birth related outcomes (Kyei-Nimakoh et al., 2017). However, the current study found wide variations in the level of maternal (ANC, heath facility delivery/skilled birth attendance, PNC, PMTCT), and child health services utilization across the region. Different factors operating at individual and household, health facility and environmental levels contribute for the variations in MNCH uptake.

The main aspects of vulnerability to unequal and poor MNCH services utilization in urban settings include poverty (Abimbola et al., 2016; Adane et al., 2017; Adewuyi et al., 2017, 2018; Aidam et al., 2005a; Anastasi et al., 2017; Asundep et al., 2013; Babirye et al., 2014; Bayou et al., 2016; Bayu et al., 2015; Belayneh et al., 2014; Bellows et al., 2012; Chege et al., 2002; Demilew, 2017; Egondi et al., 2015; Fatiregun & Okoro, 2012; Fotso et al., 2008, 2009; Idowu et al., 2017; Izugbara et al., 2009; Jennings et al., 2017; Kawakatsu et al., 2015; Kimani-Murage et al., 2015, 2016; Kuwawenaruwa et al., 2016; Machira & Palamulen, 2017; Mekonnen & Mekonnen, 2003; Mirkuzie, 2014; Mustapha et al., 2018; Mutua et al., 2011; Ndimbii et al., 2018; Ng’anjo Phiri et al., 2014; Ngandu et al., 2017; Nwameme et al., 2014; Ochola et al., 2013; Olusanya et al., 2010; Owiti et al., 2018; Rossier et al., 2014; Sasaki et al., 2010; Taffa & Chepngeno, 2005; Tann et al., 2007; Wakwoya et al., 2016; Westheimer et al., 2004; Wilunda et al., 2016; Woldeghebriel et al., 2017; Yaya et al., 2018), education (Abimbola et al., 2016; Adewuyi et al., 2018; Belayneh et al., 2014; Mekonnen & Mekonnen, 2003), younger maternal age (Adewuyi et al., 2018; Bellows et al., 2012; Fatiregun & Okoro, 2012; Mustapha et al., 2018; Mutua et al., 2011; Olusanya et al., 2010; Yaya et al., 2018), unemployment (Demilew, 2017; Olusanya et al., 2010), lower socioeconomic status and poor livelihoods (Aidam et al., 2005a, 2005b; Asundep et al., 2013; Bellows et al., 2012; Chege et al., 2002; Fotso et al., 2008, 2009; Idowu et al., 2017; Izugbara et al., 2009; Kibiribiri et al., 2016; Kimani-Murage et al., 2015, 2016; Olusanya et al., 2010), low social integration and social support (Mirkuzie, 2014), socio-cultural taboos (Adewuyi et al., 2017; Wilunda et al., 2016; Woldeghebriel et al., 2017), residing in slums (Antai, 2010; Woldeghebriel et al., 2017), being displaced (Kibiribiri et al., 2016), refugee (Kibiribiri et al., 2016), or migrant (Antai, 2010; Mirkuzie, 2014).

Evidence on inequalities in access to MNCH services based on socioeconomic gradient (i.e. wealth status or income, and education) aligns with secular findings on determinants of health in general (Adler & Newman, 2002; Bloom & Canning, 2000; Ettner, 1996; Umuhoza & Ataguba, 2018). This means the findings might not come as a surprising one. However, what this systematic review highlights is the fact that socioeconomic determinants are still largely in play and persistent in urban settings across SSA therefore contradicting the hypothesis of better economic and health prospects associated with living in cities. The reality in urban SSA as regards to health inequalities is more gloomy as predicted and evidence suggest that the so-called urban advantage has seemingly wiped out (Adler & Newman, 2002). Effective primary health care models are therefore needed to serve the needs of the less educated and poor segments of the populations, in particular women and under 5 children living in urban slums. Tackling the significant economic inequalities in access to MNCH services in urban SSA necessitates further evidence on the pathways between socioeconomic variables and access to health. One pertinent pathway is affordability of MNCH care for users. In the last decade, many international institutions including the World Health Organization (WHO) have advocated for better social (health) protection programs in the region to improve equity in access to healthcare (Sidze et al., 2020). The implementation and better targeting of such programs would benefit in understanding how affordable MNCH care is for unprivileged households.

As regards to variations by maternal age, an analysis of 34 SSA countries’ DHS data showed that older women have higher odds of facility-based delivery than younger mothers (Dunlop et al., 2018). Similar findings were also observed in a study conducted in India (Singh & Singh, 2014). The main reason for reduced level of MNCH services use among younger mothers could be low autonomy in making decision towards MNCH services access and use, as well as lower awareness towards obstetric danger signs than older women (Dunlop et al., 2018). This suggests the need of intervention programs that increase the autonomy over access and use of MNCH services utilization among young women in SSA. Particularly, age-sensitive intervention programs are needed to improve service utilization (Singh & Singh, 2014). Overall, due attention should be given to younger women to achieve UHC and thereby improve MNCH services in the region.

At health system level, facility-based health promotion activities such as counselling and support from health workers and peers (Anastasi et al., 2017), family and professional support (Kimani-Murage et al., 2015), proper instruction/counselling by health care staff (Adane et al., 2017; Chege et al., 2002; Mekonnen & Mekonnen, 2003; Wakwoya et al., 2016), and better health workers attitude (Ndimbii et al., 2018; Nwameme et al., 2014) were found to be effective in improving the observed variations in MNCH services utilization. Identifying and educating mothers whose children were at risk (Fatiregun & Okoro, 2012), improving perception about pregnancy and the benefits of institutional delivery assistance, and improving communication between health care workers and refugees and migrant populations (Kibiribiri et al., 2016) were also found as key in improving MNCH service utilization. Improving physical access to health facilities (Fotso et al., 2008), improved service availability (Asundep et al., 2013; Babirye et al., 2014; Demilew, 2017; Idowu et al., 2017; Mekonnen & Mekonnen, 2003; Ngandu et al., 2017; Owiti et al., 2018; Sasaki et al., 2010), and strengthening of health facilities (Babirye et al., 2014; Tann et al., 2007), were also identified factors that enhanced MNCH services utilization among poor communities.

## Limitations of the Review

This systematic review has some limitations. First, only studies that were conducted in urban SSA were included in this study, and we excluded studies that were conducted in rural areas for better representation of the study population. Second, this review followed the PRISMA guideline for systematic review. However, most of the studies included in this review were from the Eastern and Western African countries and some other countries/sub-regions were not represented in this review. Additionally, as this study was only a systematic review, we did not conduct a meta-analysis due to heterogeneities among studies.

## Supplementary Information

Below is the link to the electronic supplementary material.

Supplementary file1 (PDF 314 kb)

Supplementary file2 (PDF 639 kb)
