# Correction to: Inequalities in Access and Utilization of Maternal, Newborn and Child Health Services in sub‑Saharan Africa: A Special Focus on Urban Settings

**DOI:** 10.1007/s10995-022-03396-4

**Published:** 2022-03-01

**Authors:** E. M. Sidze, F. M. Wekesah, L. Kisia, A. Abajobir

**Affiliations:** African Population and Health Research Center (APHRC), APHRC Campus, 2nd Floor, Manga Close off Kirawa Road, P.O. Box 10787‑00100, Nairobi, Kenya

## Correction to: Maternal and Child Health Journal (2022) 26:250–279 10.1007/s10995-021-03250-z

The article “Inequalities in Access and Utilization of Maternal, Newborn and Child Health Services in sub‑Saharan Africa: A Special Focus on Urban Settings”, written by E. M. Sidze, F. M. Wekesah , L. Kisia, A. Abajobir was originally published electronically on the publisher’s internet portal on 15 October 2021 without open access. With the author(s)’ decision to opt for Open Choice the copyright of the article changed on 12 February 2022 to © The Author(s) 2021 and the article is forthwith distributed under a Creative Commons Attribution 4.0 International License, which permits use, sharing, adaptation, distribution and reproduction in any medium or format, as long as you give appropriate credit to the original author(s) and the source, provide a link to the Creative Commons licence, and indicate if changes were made. The images or other third party material in this article are included in the article’s Creative Commons licence, unless indicated otherwise in a credit line to the material. If material is not included in the article’s Creative Commons licence and your intended use is not permitted by statutory regulation or exceeds the permitted use, you will need to obtain permission directly from the copyright holder. To view a copy of this licence, visit http://creativecommons.org/licenses/by/4.0.

The original article has been corrected.

