# Supplementary material for: Inequalities in Access and Utilization of Maternal, Newborn and Child Health Services in sub-Saharan Africa: A Special Focus on Urban Settings
Source: Matern Child Health J. 2021 Oct 15;26(2):250–79. doi: 10.1007/s10995-021-03250-z (PMC8888372; doi:10.1007/s10995-021-03250-z)
Supplement: Supplementary file 1 — Supplementary file1 (PDF 314 kb) [file 10995_2021_3250_MOESM1_ESM.pdf]

Search strategy

**Domain:** Adults in SSA (both general population and women)	**Determinant:** Aspects of equity		**Outcome:** Access to maternal and child health services					
	**OR (1)**							
**Sub-Saharan Africa**	SES	Health equity	Healthcare accessibility	Affordability	Maternal health services	Postnatal	Skilled delivery	Child health services
**SSA Countries:** Benin, Botswana, Burkina Faso, Cameroun, Congo (Bazaville), Congo (DRC), Ethiopia, Ghana, Guinea, Ivory Coast, Kenya, Lesotho, Madagascar, Malawi, Mauritius, Mozambique, Namibia, Niger, Nigeria, Rwanda, Senegal, Sierra Leone, South Africa, Swaziland, Tanzania, Togo, Uganda, Zambia	Wealth	Healthcare disparities	Healthcare availability	Affordable	Antenatal	Postnatal care	Assisted delivery	Immuniz*
	Income				Antenatal care	PNC	Traditional birth attendant	Vaccinat*
	Education				ANC	Postpartum	TBA	Breastfeeding
					Prenatal education		Midwif*	Infant and young child nutrition
					Prenatal care		Midwiv*	
					Prenatal			

					Perinatal care			
					Supplement*			
					Medication			
**MESH TERMS**								
Africa South of the Sahara"[Mesh]	Socioeconomic Factors [Mesh]	Health Equity [Mesh]	Healthcare Disparities [Mesh]		Prenatal Education [Mesh]	Maternal Health Services [Mesh]	Delivery, Obstetric [Mesh]	"Vaccination/administration and dosage"[Majr]
			Health Services Accessibility [Mesh]				Midwifery [Mesh]	"Vaccination/therapy"[Majr]
								"Immunization/administrati

								on and dosage"[Majr]
								"Immunization/therapy"[Majr]
								"Immunization/prevention and control"[Majr]

Combinations of Terms/Keywords – PubMed

#16,"(#7 OR #15)"
#15,"(#8 AND #14)"
#14,"(#12 AND #13)"
#13,"(#9 OR #10 OR #11)"
#12,"(maternal health services OR antenatal care OR antenatal OR prenatal care OR prenatal education OR prenatal OR maternal-child health services OR postnatal care OR postpartum care OR obstetric delivery OR assisted delivery OR traditional birth attendant OR midwife OR midwives OR community health workers OR child health services OR immuniz* OR vaccinat*)"
#11,"(health equity OR healthcare disparit* OR healthcare accessibility OR healthcare availability OR affordability)"
#10,"(health equity OR health* disparit* OR healthcare accessibility OR healthcare availability OR healthcare affordability)"
#9,"(socioeconomic status OR wealth OR income OR poverty OR education)"
#8,"(Benin[Title/Abstract] OR Botswana[Title/Abstract] OR Burkina Faso[Title/Abstract] OR Cameroun[Title/Abstract] OR Cameroon[Title/Abstract] OR Congo[Title/Abstract] OR Congo, Ethiopia[Title/Abstract] OR Ghana[Title/Abstract] OR Guinea[Title/Abstract] OR Ivory Coast[Title/Abstract] OR Kenya[Title/Abstract] OR Lesotho[Title/Abstract] OR Madagascar[Title/Abstract] OR Malawi[Title/Abstract] OR Mauritius[Title/Abstract] OR Mozambique[Title/Abstract] OR Namibia[Title/Abstract] OR Niger[Title/Abstract] OR Nigeria[Title/Abstract] OR Rwanda[Title/Abstract] OR Senegal[Title/Abstract] OR Sierra Leone[Title/Abstract] OR South Africa[Title/Abstract] OR Swaziland[Title/Abstract] OR Tanzania[Title/Abstract] OR Togo[Title/Abstract] OR Uganda[Title/Abstract] OR Zambia[Title/Abstract])",261734,10:07:37

#7,"(#1 AND #3 AND #6)"
#6,"(#3 OR #4)"
#5,"(#1 AND #2 AND #3)"
#4,"((""Vaccination/administration and dosage""[Majr] OR ""Vaccination/therapeutic use""[Majr])) OR (""Immunization/administration and dosage""[Mesh] OR ""Immunization/prevention and control""[Mesh] OR ""Immunization/therapeutic use""[Mesh] OR ""Immunization/therapy""[Mesh])"
#3,"(((""Maternal Health Services""[Mesh]) OR ""Prenatal Education""[Mesh]) OR ""Delivery, Obstetric""[Mesh]) OR ""Midwifery""[Mesh]"
#2,"(((""Socioeconomic Factors""[Mesh]) OR ""Health Equity""[Mesh]) OR ""Healthcare Disparities""[Mesh]) OR ""Health Services Accessibility""[Mesh]",510000,08:45:36
#1,""Africa South of the Sahara""[Mesh] or"

Search strategy – for other databases including PopLine

maternal healthcare OR antenatal care OR postnatal care OR midwifery OR postpartum OR birth OR skilled delivery OR assisted delivery OR perinatal care OR maternal and childcare OR immunization OR vaccination OR obstetric delivery OR assisted delivery OR delivery

AND

healthcare accessibility OR healthcare disparities OR health equity OR socioeconomic status OR poverty OR wealth

AND

Africa sub Saharan

**1**	((((healthcare accessibility OR healthcare disparities OR health equity OR socioeconomic status OR poverty OR wealth) AND (maternal healthcare OR antenatal care OR postnatal care OR midwifery OR postpartum OR birth OR skilled delivery OR assisted delivery OR perinatal care OR maternal and childcare OR immunization OR vaccination OR obstetric delivery OR assisted delivery OR delivery)) AND ((Taxonomy term IDs from the <em class="placeholder">Region/Country vocabulary:Africa Sub Saharan)))) AND ((Taxonomy term IDs from the <em class="placeholder">Language vocabulary:English) AND (is_field_document_year_int:[2000 TO 2018]))
