# Supplementary material for: Inequalities in Access and Utilization of Maternal, Newborn and Child Health Services in sub-Saharan Africa: A Special Focus on Urban Settings
Source: Matern Child Health J. 2021 Oct 15;26(2):250–79. doi: 10.1007/s10995-021-03250-z (PMC8888372; doi:10.1007/s10995-021-03250-z)
Supplement: Supplementary file 2 — Supplementary file2 (PDF 639 kb) [file 10995_2021_3250_MOESM2_ESM.pdf]

Supplementary Table 1: Mixed methods approach (quantitative and qualitative)

**Author, journal, year**	**Study aim/title of study**	**Design/ Data source (Cross-sectional (quanti/ quali), longitudinal, prospective, retrospective (including health facilities record review), secondary analysis of data, case-control, RCT, survey (DHS, SPA, MICs ect)**	Was the research question or objective in this paper clearly stated?	Was the study population clearly specified and defined?	Was the participation rate of eligible persons at least 50%?	Were all the subjects selected or recruited from the same or similar populations (including the same time period)? Were inclusion and exclusion criteria for being in the study prespecified and applied	Was a sample size justification, power description, or variance and effect estimates provided?	For the analyses in this paper, were the exposure(s) of interest measured prior to the outcome(s) being measured?	Was the timeframe sufficient so that one could reasonably expect to see an association between exposure and outcome if it existed?	For exposures that can vary in amount or level, did the study examine different levels of the exposure as related to the outcome (e.g., categories of exposure, or exposure	Were the exposure measures (independent variables) clearly defined, valid, reliable, and implemented consistently across all study participants?	Was the exposure(s) assessed more than once over time?	Were the outcome measures (dependent variables) clearly defined, valid, reliable, and implemented consistently across all study participants?	Were the outcome assessors blinded to the exposure status of participants?	Was loss to follow-up after baseline 20% or less?	Were key potential confounding variables measured and adjusted statistically for their impact on the relationship between exposure(s) and outcome(s)?	**Quality rating**

						uniformly to all participants?				measured as continuous variable)?							
Birungu et al, International Perspectives on Sexual and Reproductive Health, 2011	Maternal Health Care Utilization Among HIV-Positive Female Adolescents in Kenya	Cross-sectional; Analysis of pregnancy history data						NA	NA	N/A		NA		N/A	NA		**Good**
Taffa & Chepngeno, Tropical Med Int H, 2005	Determinants of health care seeking for childhood illnesses in Nairobi slums	Survey; the Nairobi Urban Demographic Surveillance System (NUDSS).						NA	NA	N/A		NA		N/A	NA		**Good**
Chege et al, East Af. M J, 2002	Childcare practices of commercial seks workers	Cross-sectional; Mixed methods; FGDs						N/A	N/A	N/A		NA		N/A	NA		**Fair**

Tann et, BMC Preg Childbirth, 2007	Use of antenatal services and delivery care in Entebbe, Uganda: a community survey	cross-sectional retrospective community survey						NA	NA	N/A		NA		N/A	NA		**Good**
Sanni et al, Int Health, 2018	Factors associated with maternal utilization of health facilities for delivery in Ethiopia	Cross-sectional; 2011 DHS						NA	NA	N/A		NA		N/A	NA		**Good**
Ngandu et al, BMJ Open, 2017	Wealth-related inequality in early uptake of HIV testing among pregnant women: an analysis of data from a national cross-	Cross-sectional survey, 2012 PMTCT programme						N/A	N/A	N/A		NA		N/A	NA		**Good**

	sectional survey																
Mustapha et al, BMC infectious diseases, 2018	Utilization of “prevention of mother-tochild transmission” of HIV services by adolescent and young mothers in Mulago Hospital, Uganda	cross-sectional and mixed methods study which employed both quantitative and qualitative methods						N/A	NA	NA		NA		N/A	NA		**Fair**
Anastasi et al, PLOS ONE, 2017	Unmasking inequalities: Sub-national maternal and child mortality data from two urban slums in Lagos,	Cross-sectional, community-based household survey						NA	NA	NA		NA		N/A	NA		**Fair**

	Nigeria tells the story															
Adewuyi et al, Scandinavian journal of Public health, 2017	Prevalence and factors associated with non-utilization of healthcare facility for childbirth in rural and urban Nigeria: Analysis of a national population-based survey	Cross-sectional survey						NA	NA	NA		NA		N/A	NA		**Good**
Antai, BMC Public Health, 2010	Migration and child immunization in Nigeria: individual- and community-level factors	2003 Nigeria Demographic and Health Survey (DHS)						NA	NA	NA		NA		N/A	NA		**Good**

Mekonnen & Mekonnen, J Health Popul Nutr, 2003	Factors Influencing the Use of Maternal Healthcare Services in Ethiopia	2000 Ethiopia Demographic and Health Survey						NA	NA	NA		NA		N/A	NA		**Fair**
Owiti et al, Pan African Medical Journal, 2018	Utilization of Kenya's free maternal health services among women living in Kibera slums: a cross-sectional study	Cross-sectional						NA	NA			NA		N/A	NA		**Good**
Adewuyi et al, PLOSONE, 2018	Prevalence and factors associated with underutilization of antenatal care services in Nigeria: A	2013 Nigeria Demographic and Health Survey						NA	NA	NA		NA		N/A	NA		**Good**

	comparative study of rural and urban residences based on the 2013 Nigeria demographic and health survey																
Kawakatsu et al, Public health, 2015	Effects of three interventions and determinants offull vaccination among children aged 12-59months in Nyanza province, Kenya	cross-sectional study using the Multiple Indicator Cluster Survey (MICS) 2011 conducted by the Kenya National Bureau of Statistics (KNBS) with support from UNICEF					NR	N/A	N/A	N/A		N/A		N/A	NA		**Fair**

Machira and Palamuleni, Journal of Egypt Public Health Association, 2017	The factors associated with women's use of postpartum care services in urban areas as compared with the rural areas in Malawi.	2010 Malawi Demographic and Health Survey data which is the national representative survey of women in the reproductive age between 15 and 49 years.						N/A	N/A	N/A		NA		N/A	NA		**Good**
Engodi et al, IJEH, 2015	Determinants of immunization inequality among urban poor children: evidence from Nairobi's informal settlements	Nairobi Cross-Sectional Slum Survey of 2012					NR	N/A	N/A	N/A	NR	N/A	NR	N/A	NA		**Fair**

Phiri et al, BMC Preg Childbirth, 2014	Factors associated with health facility childbirth in districts of Kenya, Tanzania and Zambia: a population based survey	A population-based survey in 2007						N/A	N/A	N/A		N/A		N/A	NA		**Fair**
Fotso, Ezeh & Oronje; Journal of Urban Health, 2008	Describe the provision of obstetric care in the Nairobi informal settlements; (2) describe the patterns of antenatal and delivery care, notably in terms of	Cross-sectional						N/A	N/A	N/A	NR	N/A	NR	N/A	NA		**Good**

	timing, frequency, and quality of care;																
Fotso et al, Maternal and child health Journal, 2008	Identify the factors which influence the choice of place of delivery among the urban poor in Nairobi, Kenya	Cross-sectional						N/A	NA	NA	NA	NA		N/A	NA		**Good**
Bayu et al, Global Health Action, 2015	Identify factors affecting unplanned home delivery in urban settings	Community-based follow-up study										NA		N/A	N/A		**Fair**
Kibiribiri et al, Int J of Gyn & Ob, 2016	Examine disparities in the quality of prenatal care received by	A cross-sectional, mixed methods study					NR	N/A	N/A	N/A		N/A		N/A	N/A		**Fair**

	pregnant refugee women and local South African pregnant women																
Aidam et al EJCN, 2005	Assess factors associated with exclusive breast-feeding (EBF) in Accra, Ghana	Cross-sectional					NR	N/A	CD	N/A		N/A		N/A	N/A		**Fair**
Bellows et al, Health Policy Plan, 2012	Measure the association between the introduction of an output-based voucher programme and the odds of a facility-based delivery in two Nairobi informal	Cross-sectional analysis of the 2004 - 2005 & 2006–2008 NUDHSS data					RNR	N/A	N/A	N/A		N/A		N/A	N/A		**Fair**

	settlements.																
Olusanya, Alakija, Inem, J Biosc Sci, 2010	Etablish the pattern and uptake of maternity services and associated factors against the backdrop of rapid urbanization in Nigeria.	Cross-sectional					N/R**	N/A	N/A	N/A		N/A		N/A	N/A		**Good**
Rossier et al, Global Health Action, 2014	To assess MH utilization in Nairobi and Ouagadougou	Cross-sectional analysis of 2009 - 2011 HDSS data					NR	N/A	N/A	N/A		N/A		N/A	N/A		**Good**
Belayneh, Adefris and Andargie, 2014	Assess timing of ANC booking and associated factors	Hospital-based cross-sectional study						N/A	N/A	N/A		N/A		N/A	N/A		**Fair**

	among pregnant women attending ANC clinic at University of Gondar Hospital, 2013																
Babirye et al, BMC Health Services Research 2014	Urban settings do not ensure access to services: findings from the immunisation programme in Kampala Uganda	Mixed methods approach (quantitative and Qualitative collected)						N/A	N/A	N/A		N/A		N/A	N/A		**Good**
Fatiregun et al, Vaccine, 2011	Maternal determinants of complete child immunization among children aged 12–23 months in a southern	A cross sectional study design						N/A	N/A	N/A		N/A		N/A	N/A		**Good**

	district of Nigeria																
Idowu et al., Ethiopian journal of health sciences, 2017	Determinants of Skilled Care Utilization among Pregnant Women Residents in an Urban Community in Kwara State, Northcentral Nigeria	A cross sectional study design						N/A	N/A	N/A		N/A		N/A	N/A		**Good**
Demilew, BMC Res Notes, 2017	Factors associated with mothers' knowledge on infant and young child feeding recommendation in slum areas of Bahir	A cross sectional study design						N/A	N/A	N/A		N/A		N/A	N/A		**Good**

	Dar City, Ethiopia: cross sectional study																
Adane et al. Journal of Health, Population and Nutrition, 2017	Utilization of health facilities and predictors of health-seeking behavior for under-five children with acute diarrhea in slums of Addis Ababa, Ethiopia: a community-based cross-sectional study	A cross sectional study design						N/A	N/A	N/A		N/A		N/A	N/A		**Good**
Abimbola et al, African health	Pattern of utilization of ante-natal and	A descriptive cross-sectional study						N/A	N/A	N/A		N/A		N/A	N/A		**Fair**

sciences, 2016	delivery services in a semi-urban community of North-Central Nigeria.																
Wakwoya et al, Pan African Medical journal, 2016	Infant feeding practice and associated factors among HIV positive mothers in Debre Markos Referral Hospital East Gojam zone, North West Ethiopia	A cross sectional study design						N/A	N/A	N/A	N/R	N/A	N/R	N/A	N/A		**Good**
Kuwawenaruwa et al. Journal of Health, Population and Nutrition, 2016	The effects of MCH insurance cards on improving equity in access and use of	Mixed methods approach (quantitative and Qualitative)					NR	N/A	N/A	N/A		N/A		N/A	N/A		**Fair**

	maternal and child health care services in Tanzania: a mixed methods analysis																
Woldegh ebriel et al., Maternal and child nutrition, 2017	Length of time in Ghana is associated with the likelihood of exclusive breastfeeding among Liberian refugees living in Buduburam	Cross-sectional study					NR	N/A	N/A	N/A		N/A		N/A	N/A		**Goo d**
Bayou et al. BMC Pregnancy and Childbirth, 2016	The adequacy of antenatal care services among slum residents in Addis Ababa, Ethiopia	Cross-sectional design						N/A	N/A	N/A		N/A		N/A	N/A		**Goo d**

Sasaki et al, Tropical Medicine and International Health, 2010	Access to a health facility and care-seeking for danger signs in children: before and after a community-based intervention in Lusaka, Zambia	Repeated Cross-sectional design					NR	N/A	N/A	N/A				N/A	N/A		**Good**
Westheimer et al, Journal of Acquired Immune Deficiency Syndrome, 2004	Acceptance of HIV Testing Among Pregnant Women in Dar-es-Salaam, Tanzania	Cross-secttional design					NR	N/A	N/A	N/A		N/A		N/A	N/A		**Good**
Nwameme et al, Maternal Child Health Journal, 2013	Compliance with Emergency Obstetric Care Referrals Among Pregnant Women	Cross-sectional study						N/A	N/A	N/A		N/A		N/A	N/A		**Fair**

	in an Urban Informal Settlement of Accra, Ghana																
Asundep et al,Journal of Epidemiology and Global Health, 2013	Determinants of access to antenatal care and birth outcomes in Kumasi, Ghana	Cross-sectional study					NR	N/A	N/A	N/A		N/A		N/A	N/A		**Good**
Sasaki et al, J Epid & Comm Health, 2019	To examine the association between immunisation coverage and distance to an immunisation service as well as socio-demographic and economic	Repeated cross-sectional surveys					NR			N/A				N,A			**Good**

	factors before and after the introduction of outreach immunisation services, and to identify optimal locations for outreach immunisation service points in a peri-urban area in Zambia																
Mutua et al, BMC Public Health, 2011	Childhood vaccination in informal urban settlements in Nairobi, Kenya: Who gets vaccinated?	Longitudinal											N/R			**Good**

Jennings et al., Maternal and Child Health Journal 2017	Association of Household Savings and Expected Future Means with Delivery Using a Skilled Birth Attendant in Ghana and Nigeria: A Cross-Sectional Analysis	A multi-country longitudinal study						N/A	N/A	N/A		N/A		N/A	N/A		**Good**
Kimani-Murage et al, Int J Dohad, 2016	Determine potential effectiveness of the national Community Health Strategy involving home-based counselling visits by	A quasi-experimental study. Data from -a longitudinal observational study												N/A	N/A		**Good**

	CHWs on EBF																
Fotso, Ezeh & Esendi, 2009, Rep Health	Maternal health in resource-poor urban settings: how does women's autonomy influence the utilization of obstetric care services	Cross-sectional						N/A	N/A	N/A							**Good**
Govender, SA Fam Practice, 2018	Obstetric outcomes and antenatal access among adolescent pregnancies in KwaZulu-Natal, South Africa	Mixed methods					NR	N/A	N/A	N/A		N/A		N/A	N/A		**Fair**

Supplementary Table 2: Qualitative approach

**Author, journal, year**	**Study aim/title of study**	**Design/Data source (Cross-sectional (quanti/quali), longitudinal, prospective, retrospective (including health facilities record review), secondary analysis of data, case-control, RCT, survey (DHS, SPA, MICs ect)**	Was there clear statement of aims of the research?	Is the qualitative methodology appropriate?	Was the research design appropriate to address aims of the research?	Was the recruitment strategy appropriate to the aims of the research?	Was the data collected in a way that address the research issue?	Has the relationship between researcher and participants been adequately considered?	Has ethical issues been taken into consideration?	Was the data analysis sufficiently rigorous?	Is there a clear statement of findings?	How valuable is the research	**Quality rating**
Ndimbii et al, Sexual & Reproductive Healthcare, 2018	Access and utilisation of reproductive, maternal, neonatal and child health services among women who inject drugs in coastal Kenya	Qualitative focus group discussion and indepth interviews											**Good**
Kimani-Murage et al,Mat Ch Nut, 2015	Investigates the factors that contribute to poorbreastfeeding practices in two urban slums in Nairobi,Kenya, using	Qualitative: 19 in-depth interviews (IDIs),10 focus group discussions (FGDs) and 11 key infor-mant interviews (KIIs) in April 2012											**Good**

	qualitative methods												
Izugbara, Kabiru, Zulu, PH reports, 2009	To assess the views of poor urban women on the attractions of and deterrents to hospital-based deliveries	Qualitative - 12 FGDs											**Fair**
Wilunda et al, PLOSONE, 2016	Barriers to Institutional Childbirth in Rumbek North County, South Sudan: A Qualitative Study	Qualitative											**Good**
Mirkuzie, IJEH, 2014	Explored the inequities in maternal health care among migrant women in Addis Ababa, Ethiopia	Community based qualitative study											**Good**

Supplementary Table 3: RCT

**Author, journal, year**	**Study aim/title of study**	**Design/Data source (Cross-sectional (quanti/quali), longitudinal, prospective, retrospective (including health facilities record review), secondary analysis of data, case-control, RCT, survey (DHS, SPA, MICs ect)**
Ochola, Labadarios, Nduati, Pub Health Nutri, 2013	Determine the impact of facility-based semi-intensive and home-based intensive counselling in improving exclusive breast-feeding (EBF) in a low-resource urban setting in Kenya	Cluster randomized controlled trial April 2006 to April 2008:
Aidam , Pérez-Escamilla, Lartey, J Nutr, 2005	Determine the effect of lactation counseling on EB	RCT: Intervention groups (IG) 1 - EBF support given pre-, peri-, and postnatally (IG1; n = 43); 2) EBF support given only peri- and postnatally (IG2; n = 44); or CG - nonbreast-feeding health educational support (n = 49)

**Studies with pre-protocol**	Unique ID	Study ID	Experimental	Comparator	Outcome		Randomization process	Deviations from intended interventions	Missing outcome data	Measurement of the outcome	Selection of the reported result	Overall				
	R1	RCT	exclusive breastfeeding counselling	usual health education	exclusive breastfeeding	Ochola et al 21012	+	+	+	?	?	!			+ Low risk	
	R2	RCT2	Counselling	Usual care	Exclusive breastfeeding	Aidam et al 2005	+	+	+	+	?	+			? Some concerns	
															− High risk	
